# Safety and efficacy of angiotensin receptor neprilysin inhibitor in improving cardiac function and blood pressure in dialysis patients

**DOI:** 10.3389/fmed.2024.1421085

**Published:** 2024-09-05

**Authors:** Kai Zhou, Qiuyue Zhang, Wen Dong, Xin Li, Yimiao Sun, Ying Zhang

**Affiliations:** ^1^Xuzhou Medical University, Xuzhou, China; ^2^The Affiliated Hospital of Xuzhou Medical University, Xuzhou, Jiangsu, China

**Keywords:** angiotensin receptor neprilysin inhibitor, cardiac function, blood pressure, dialysis patients, left ventricular function

## Abstract

**Background:**

The efficacy of the angiotensin receptor neprilysin inhibitor (ARNI) sacubitril/valsartan (SV) in patients with chronic kidney disease (CKD) has been established. Two meta-analyses have demonstrated its significant role in enhancing ventricular remodeling. However, the effectiveness and safety of its use in patients with end-stage renal disease (ESRD) remain unclear.

**Methods and results:**

Up to October 2023, we searched the PubMed, Embase, and Web of Science databases for studies involving ESRD patients treated with ARNI. The quality of the included studies was evaluated using the Newcastle-Ottawa Scale. Effect sizes were reported as mean differences (MD) with 95% confidence intervals (CIs). We included 10 studies, encompassing 649 patients. ARNI was associated with improvements in blood pressure and left ventricular (LV) function in ESRD patients, including systolic blood pressure (SBP) (MD −12.76 mmHg; 95% CI, −18.03 to −7.5 mmHg), diastolic blood pressure (DBP) (MD −6.41 mmHg; 95% CI, −8.10 to −4.72 mmHg), and left ventricular ejection fraction (LVEF) (MD, 4.61%; 95% CI, 1.78%−7.44%). Hemoglobin levels improved, but there were no significant statistical differences in other biomarkers for dialysis. Sacubitril/valsartan was generally well tolerated in ESRD patients. Improved indices of left ventricular function were noted at 6 months and were more pronounced at 12 months. A linear relationship between LVEF and left ventricular end-diastolic volume (LVEDV) was observed, as indicated by a high correlation coefficient (r-value).

**Conclusion:**

ARNI effectively reduces blood pressure and enhances left ventricular function in dialysis patients, with early treatment associated with greater benefits. ARNI also demonstrates a favorable safety profile in this population. Further prospective studies are required to fully understand the long-term efficacy and safety of sacubitril/valsartan in dialysis patients.

## Introduction

In recent years, the number of individuals on dialysis has been increasing annually (1). Cardiovascular disease (CVD) is the predominant cause of mortality in patients with end-stage kidney disease (ESKD), with cardiovascular mortality rates being 10–20 times higher in dialysis patients compared to age-matched individuals from the general population (2–4). Both hemodialysis and peritoneal dialysis patients require comprehensive management beyond standard dialysis treatment, and improving cardiac function is a critical concern.

Angiotensin receptor neprilysin inhibitor (ARNI) is a novel therapeutic agent that concurrently inhibits the renin-angiotensin-aldosterone system (RAAS) and enkephalinase and augments the natriuretic peptide system. This dual action results in cardioprotective effects such as natriuresis, vasodilation, and reversal of ventricular remodeling (5–8). Previous research has demonstrated the superiority of ARNI over angiotensin II receptor antagonists or angiotensin-converting enzyme inhibitors in improving myocardial remodeling, controlling heart failure, and preserving residual kidney function in non-dialyzed patients with chronic kidney disease (CKD). Moreover, meta-analyses have highlighted its distinctive role in ameliorating ventricular remodeling (9, 10). The 2019 KDIGO conference consensus paper lists ARNI as a foundational medication for treating heart failure with reduced ejection fraction (HFrEF) in the context of CKD and considers its use in dialysis patients with HFrEF (11).

The PARADIGM-HF trial noted no significant differences in serum creatinine levels between the ARNI and enalapril groups at 8- and 27-months post-randomization. However, ARNI demonstrated a stronger hypotensive effect on renal perfusion, and fewer patients in the ARNI group discontinued the study due to renal complications than those treated with enalapril. Despite these findings, the PARADIGM-HF and PARAGON-HF trials excluded patients with an estimated glomerular filtration rate (eGFR) of <30 ml/min/1.73 m^2^ (12, 13).

While the majority of the existing studies focus on the CKD population, several have shown the benefits of ARNI for cardiac function in dialysis patients. Nevertheless, some studies suggest that ARNI does not confer significant benefits in this group, rendering the effectiveness of ARNI in dialysis patients as yet inconclusive. Against this backdrop, we conducted this meta-analysis to evaluate the impact of ARNI on blood pressure, cardiac function, and biomarkers in patients with end-stage renal disease.

1. What is Known?

In heart failure patients undergoing maintenance dialysis, ARNI is recommended to enhance reverse remodeling of the left ventricle, manage heart failure symptoms, safeguard residual renal function, and reduce the risk of cardiovascular events.

2. What is New?

To the best of our knowledge, this is the first meta-analysis to directly evaluate the impacts of the angiotensin receptor neprilysin inhibitor sacubitril/valsartan (SV) on blood pressure and left ventricular (LV) function in dialysis patients.

3. What Are the Clinical Implications?

The findings of our meta-analysis indicate that ARNI is effective in managing refractory hypertension in dialysis patients and may enhance reverse remodeling of the left ventricle, particularly in patients undergoing dialysis for extended periods of time. Furthermore, the earlier dialysis patients begin treatment with angiotensin receptor neprilysin inhibitors, the greater the potential benefits.

## Methods

This meta-analysis followed the Preferred Reporting Items for Systematic Reviews and Meta Analyses (PRISMA) guidelines (14). The data supporting the findings are available from the corresponding author upon reasonable request.

### Study selection

Study inclusion criteria were as follows: (1) participants (P): adult patients (aged >18 years) with end-stage renal disease (ESRD); (2) intervention (I): patients assigned to ARNI treatment; (3) comparison (C): self-comparison or other pharmacological treatments; (4) outcome (O): patients with baseline and follow-up data for at least one LV function index, measured by echocardiography, with a follow-up period of at least 3 months; and (5) study design (S): any form of observational study and clinical trial (both randomized and non-randomized).

Case reports, letters, comments, series review articles, meta-analyses, guides, animal experiments, and studies with fewer than 10 patients were excluded from the meta-analysis.

### Information sources and search strategy

Two authors (KZ and QZ) independently conducted a systematic search of PubMed, Embase, and Web of Science databases. Search terms included “sacubitril-valsartan,” “angiotensin receptor-neprilysin inhibitor,” “Renal Dialysis,” and “End-Stage Kidney Disease,” among other relevant terms (see Supplementary material S1 for the full list). The search was restricted to articles with no language limitations. Additionally, we reviewed the reference lists of included studies to identify further eligible studies not initially retrieved by our search. All citations were managed using Endnote Reference Manager version X9 (Clarivate Analytics).

### Data extraction

Data extraction was independently conducted by two authors (KZ and QZ). Any discrepancies were resolved through consultation with a third author (YZ). The extracted data included the first author's name, publication year, country, study design, treatments in control groups, sample size, and patient demographics (age, sex, dialysis modality, follow-up duration, and duration of dialysis). Three key indices were extracted: blood pressure, left ventricular function, and biomarkers relevant to dialysis, both at baseline and follow-up.

We focused on indices representing left ventricular (LV) function, which included parameters of LV systolic function (left ventricular ejection fraction [LVEF], left ventricular end-systolic volume [LVESV], left ventricular end-diastolic volume [LVEDV], left ventricular end-diastolic diameter [LVEDd], and left ventricular end-systolic diameter [LVESd]); parameters of LV diastolic function (the ratio of early mitral inflow velocity to mitral annular early diastolic velocity [E/e' ratio], peak tricuspid regurgitation velocity [peak TR Vel], and left atrial dimension [LAD]); and parameters of LV hypertrophy (left ventricular posterior wall thickness [LVPWT] and interventricular septum thickness in diastole [IVSd]). For blood pressure, we selected systolic blood pressure (SBP) and diastolic blood pressure (DBP). For biomarkers of dialysis, we selected N-terminal proB-type natriuretic peptide (NT-proBNP), hemoglobin, potassium, creatinine, calcium, phosphorus, intact parathyroid hormone (iPTH), and 24-h urine volume.

### Risk of bias

Two independent authors assessed the risk of bias and quality of the included studies using the Newcastle-Ottawa Scale for observational studies. We assessed the following three items: selection of cohort (0–4 stars), comparability (0–2 stars), and outcome (0–3 stars), with overall scores of <5 stars, 5 to 7 stars, and >7 stars indicating high, moderate, and low risk of bias, respectively (15).

### Outcome measures

The main outcomes were changes in blood pressure (SBP and DBP), parameters of LV systolic function (LVEF, LVEDV, LVEDV, LVDd, and LVDs), parameters of LV diastolic function (E/e' ratio, peak TR Vel, and LAD), parameters of LV hypertrophy (LVPWT and IVSd), and biomarkers for dialysis during the follow-up period. These indexes were all continuous variables, primarily expressed as mean ± SD.

### Statistical analysis

Data entry and analysis were conducted using Excel (Microsoft) and Review Manager (RevMan) software version 5.4. The STATA software was used to evaluate publication bias and execute sensitivity analysis. Continuous outcomes were analyzed using the mean difference (MD) and dichotomous outcomes were analyzed using the risk ratio (RR), both presented with their respective 95% confidence intervals. Heterogeneity between studies was evaluated using the Q statistic and quantified with the I^2^ test, with an I^2^ value of >50% indicating significant heterogeneity, and we used a random-effects model. The influence of individual studies on the overall effect size was examined by sensitivity analysis, employing the leave-one-out approach.

Subgroup analyses were performed based on mean age (≥60 or <60 years), dialysis modality (PD or HD), duration of follow-up (≥6 or <6 months), duration of dialysis (≥36 or <36 months), LVEF (≥50 or <50%), sample size (≥50 or <50), and study design (prospective or retrospective). Publication bias was assessed using Egger's regression tests and visual inspection of funnel plots (20, 21). A *p*-value of <0.05 was considered statistically significant.

The relationship between changes in the LV system and left ventricular function was also explored. First, the Shapiro–Wilk test was used to determine the normality of the data distribution. Depending on the distribution, Pearson's or Spearman's correlation was used for analysis. These analyses were performed using SPSS software version 26.

## Results

**Figure 1:**
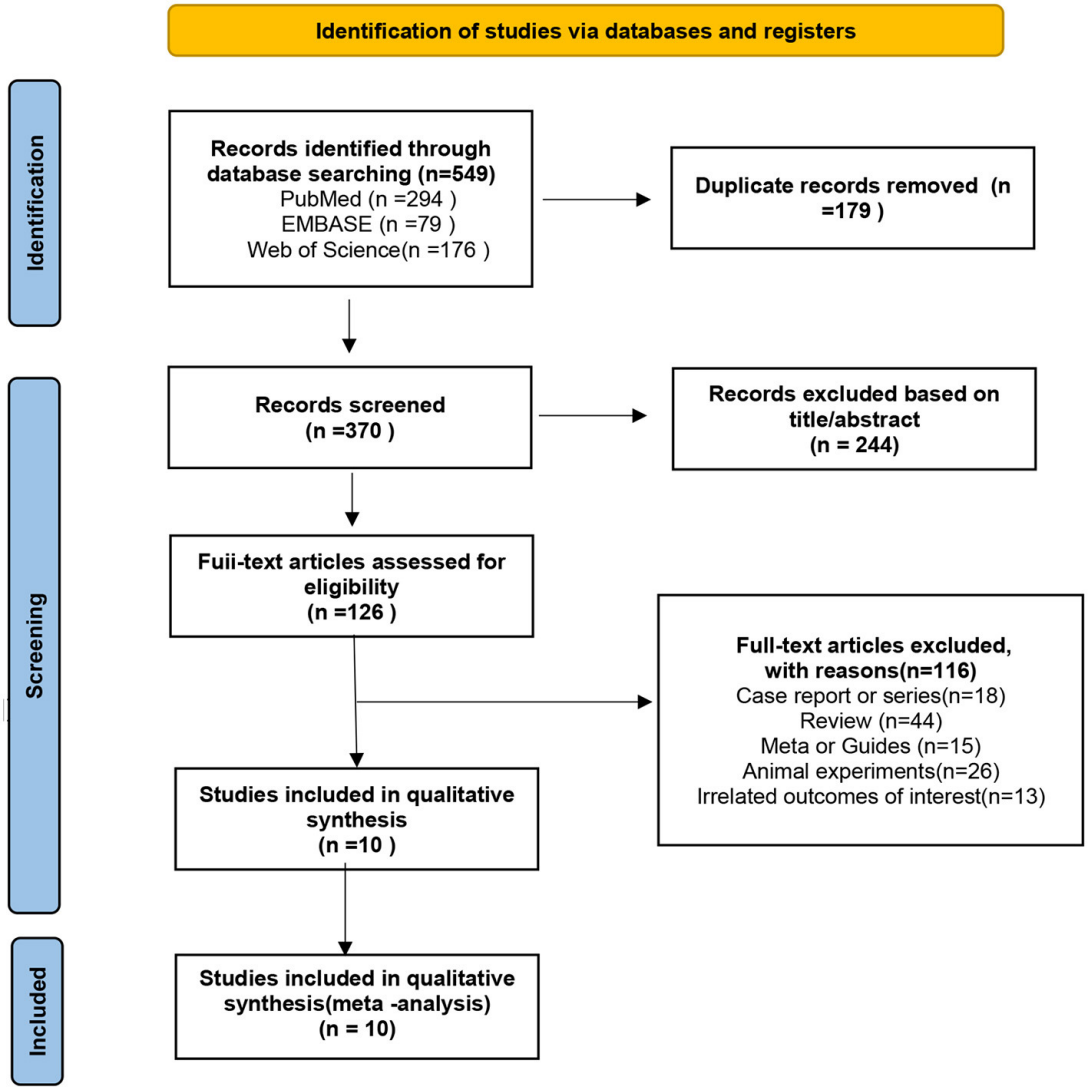
Preferred reporting items for systematic reviews and meta-analyses (PRISMA) flow diagram showing a detailed study selection process.

As of October 2023, our literature search yielded 549 publications. After removing 179 duplicates, we screened the titles and abstracts of 370 records for eligibility. Subsequently, 360 articles were excluded, resulting in the inclusion of 10 studies for both quantitative and qualitative analyses, comprising a total of 649 patients, all from observational studies. The literature search process is detailed in the Preferred Reporting Items for Systematic Reviews and Meta-Analyses flowchart shown in Figure 1.

The baseline characteristics of the studies are presented in Table 1. Publication years ranged from 2019 to 2023. The mean age of the included patients was 51.6 years, with 69.1% being men. All subjects were patients with end-stage kidney disease. The mean follow-up duration varied between 3 and 12 months. Of the 10 studies included, three (16, 18, 25) were prospective, and seven (17, 19–24) were retrospective. Four studies (16, 20, 22, 23) included a control group that received either an angiotensin receptor neprilysin inhibitor or an angiotensin-converting enzyme inhibitor, while the others lacked a control group. However, one article did not provide specific data; thus, we extracted experimental group data from three studies.

**Table 1:** Characteristics of included studies and meta-analysis patients.

**References**	**Diabetes (%)**	**Hemodialysis (%)**	**Men (%)**	**Dialysis vintage (months)**	**Follow-up (months)**
Niu et al. (16)	46.2	61.5	73.1	59.0 ± 58.2	12
Lee et al. (17)	47.8	NR	87.0	72 ± 54	6
Wang et al. (18)	44.4	100	83.3%	40.9 ± 47.4	3
Feng et al. (19)	NA	100	45.5	38.6 ± 39.3	10.5
Ding et al. (20)	62.7	37.3	68.6	29.7 ± 25.6	12
Fu et al. (21)	23.8	0	66.6	14.9 ± 3.5	NA
Ma et al. (22)	37.7	0	72.1	10.5 ± 13.7	12
Zhao et al. (23)	24	100	63%	24.2 ± 20.4	3
Guo et al. (24)	36.0	100	62.4	27.9 ± 22	8.5
Fu et al. (25)	NA	100	NR	NR	12
**References**	**Study design**	**Interventions and controls**	**Patients(n)**	**Age (y, mean** ±**SD)**	**Hypertension (%)**
Niu et al. (16)	Observational study (prospective)	ARNI ACEI/ARB	26	61.0 ± 12.1	88.5
Lee et al. (17)	Observational study (retrospective)	ARNI	23	60.9 ± 17.1	78.3
Wang et al. (18)	Observational study (prospective)	ARNI	18	53.6 ± 14.5	100
Feng et al. (19)	Observational study (retrospective)	ARNI	11	53.1 ± 16.8	NR
Ding et al. (20)	Observational study (retrospective)	ARNI ACEI/ARB	51	59.7± 13.7	100
Fu et al. (21)	Observational study (retrospective)	ARNI	21	51.0± 18.3	100
Ma et al. (22)	Observational study (retrospective)	ARNI ACEI/ARB	61	52.0 ± 13	NR
Zhao et al. (23)	Observational study (retrospective)	ARNI ACEI/ARB	71	49.1 ± 15.4	97
Guo et al. (24)	Observational study (retrospective)	ARNI	247	45.8 ± 13.7	100.0
Fu et al. (25)	Observational study (prospective)	ARNI	120	57.4 ± 15.2	NR

ACEI indicates angiotensin-converting enzyme inhibitor; ARB, angiotensin receptor blocker; ARNI, angiotensin receptor neprilysin inhibitor; NR, not reported.

### Risk-of-bias assessment

The Newcastle-Ottawa Scale scores of the included studies, which is presented in Supplementary Table S1, ranged from 6 to 9. The majority of studies (17–19, 21, 24, 25) exhibited a moderate risk of bias due to the absence of a control group. The seven retrospective studies (17, 19–24) showed a higher risk of bias. No information was provided indicating that the baseline comparisons were imbalanced. No significant publication bias was indicated by the funnel plot (Supplementary Figure S1).

### Effects of sacubitril/valsartan on blood pressure and NT-proBNP

**Figure 2:**
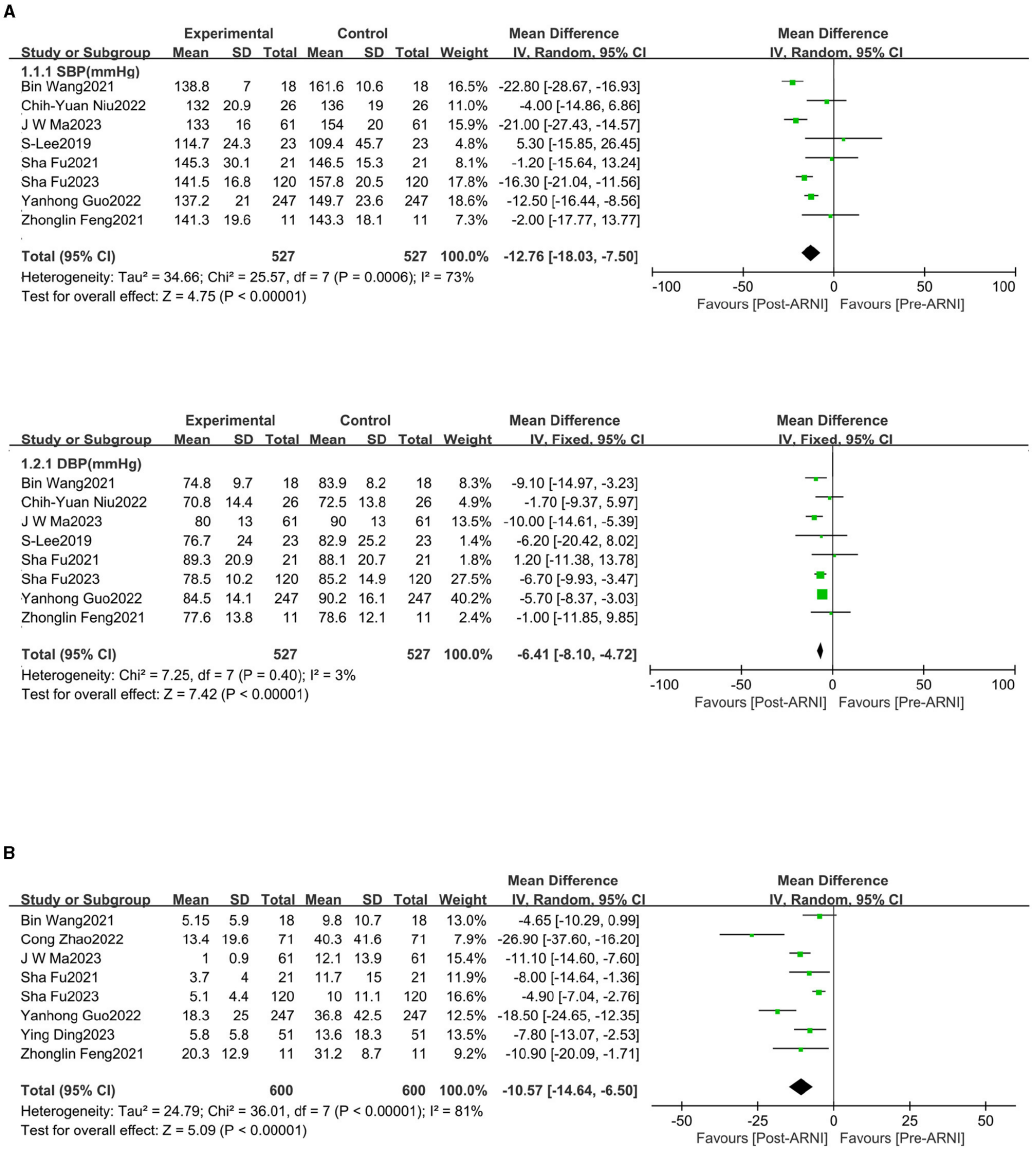
Forest plots showing changes in systolic blood pressure (SBP), diastolic blood pressure (DBP) **(A)**, and N-terminal pro-B-type natriuretic peptide (NT-proBNP) **(B)**.

Data on changes in systolic blood pressure (SBP) and diastolic blood pressure (DBP) were available from eight trials (16–19, 21, 22, 24, 25), involving 527 patients. We observed significant reductions in SBP (MD, −12.76 mmHg; 95% CI, −18.03 to −7.50 mmHg; Figure 2A and Supplementary Table S2) and DBP (MD, −6.41 mm Hg; 95% CI, −8.10 to −4.72 mmHg; Figure 2A and Supplementary Table S2). A total of eight studies (18–25) involving 600 patients reported data on NT-proBNP. The mean NT-proBNP decreased by −10.57 ^*^ 10^3^ ng/dL (95% CI, −14.64 to −6.50 ^*^ 10^3^ ng/dL; Figure 2B and Supplementary Table S2).

### Effects of sacubitril/valsartan on left ventricular systolic function

**Figure 3:**
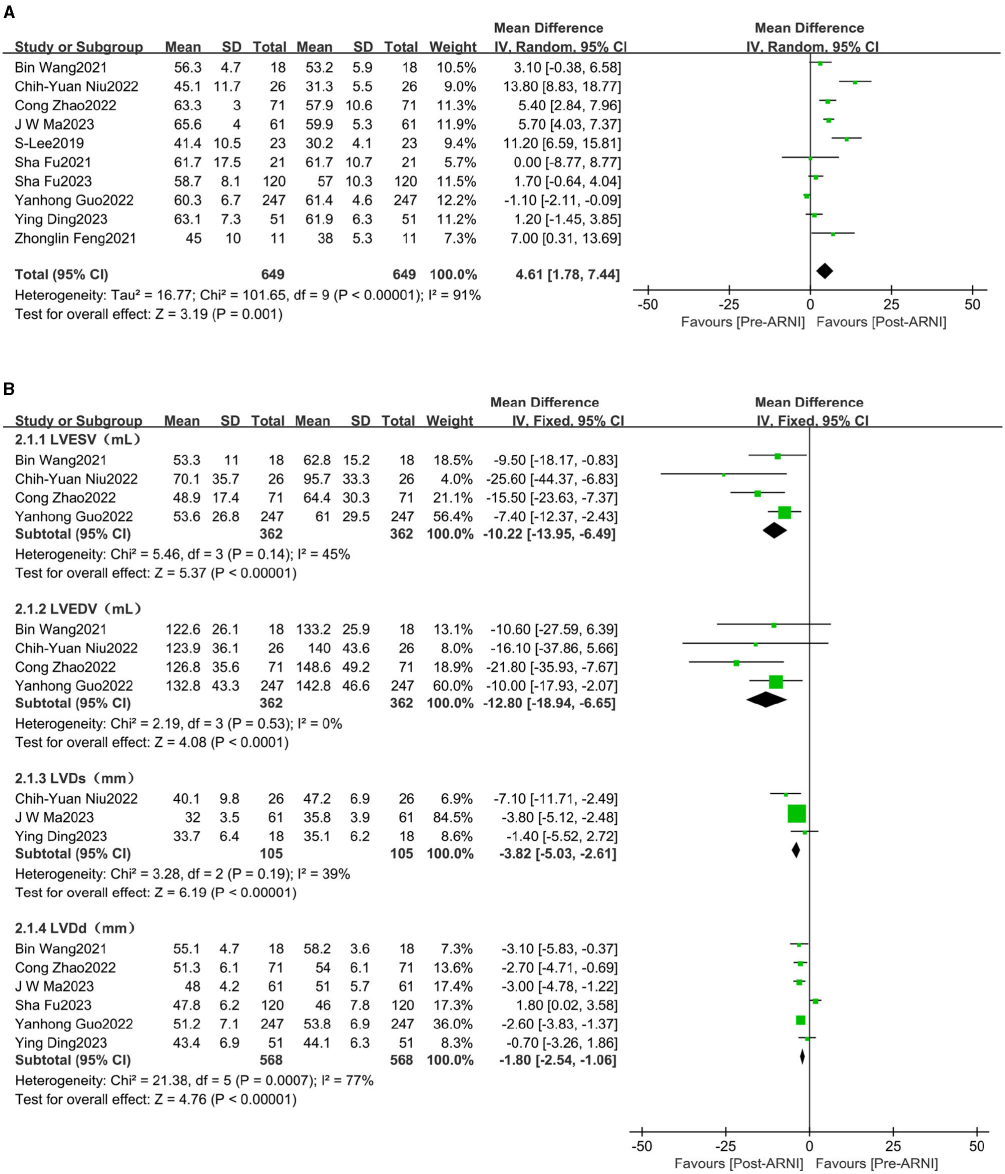
Forest plots showing changes in left ventricular ejection fraction (LVEF) **(A)**, left ventricular end-systolic volume (LVEDV), left ventricular end-diastolic volume (LVEDV), left ventricular end-diastolic diameter (LVDd), and left ventricular end-systolic diameter (LVDs) **(B)**.

The pooled data from 10 studies (16–25), involving 649 patients, indicated increased left ventricular ejection fraction (LVEF) (MD, 4.61%; 95% CI, 1.78%−7.44%; Figure 3A and Supplementary Table S3). Four studies (16, 18, 23, 24), with 362 patients, reported data on left ventricular end-diastolic volume (LVEDV). The mean LVEDV decreased by 12.80 mL (95% CI, −18.94 to −6.65). Left ventricular end-systolic volume (LVESV) was reported in four studies (16, 18, 23, 24), involving 362 patients, and was significantly decreased following treatment with sacubitril/valsartan (MD, −10.22 mL; 95% CI, −13.95 to −6.49; Figure 3B and Supplementary Table S3). Similarly, left ventricular end-systolic diameter (LVDs) (MD −3.82 mm, 95% CI −5.03 to −2.61; Figure 3B) and left ventricular end-diastolic diameter (LVDd) (MD −1.80 mm, 95% CI −2.54 to −1.06; Figure 3B) were all significantly reduced.

### Effects of sacubitril/valsartan on left ventricular diastolic function

**Figure 4:**
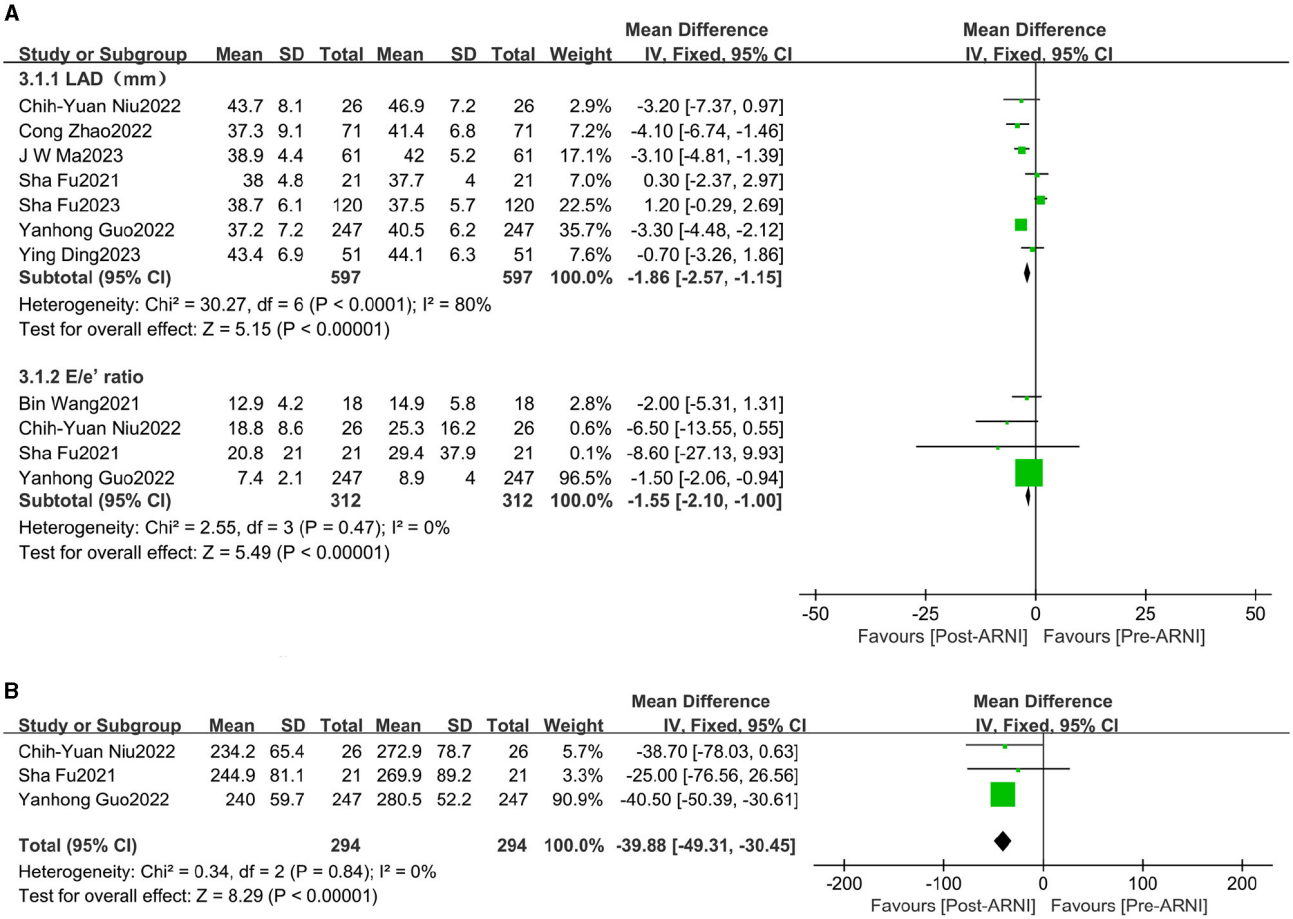
Forest plots showing changes in left atrial dimension (LAD), the ratio between early mitral inflow velocity and mitral annular early diastolic velocity (E/e' ratio) **(A)**, and peak tricuspid regurgitation velocity (peak TR Vel) **(B)**.

The pooled data from seven studies (16, 20–25), involving 597 patients, showed increases in left atrial dimension (LAD) (MD, −1.86 mL; 95% CI, −2.57 to −1.15; Figure 4A and Supplementary Table S3). Four studies (16, 18, 21, 24), comprising 312 patients, reported data on the E/e' ratio. The mean E/e' ratio decreased by 1.55 (95% CI, −2.10 to −1.00; Figure 4A and Supplementary Table S3). Three studies (16, 21, 24), involving 294 patients, reported data on peak tricuspid regurgitation velocity (Peak TR Vel), which improved following treatment with sacubitril/valsartan (MD, −39.88 cm/s; 95% CI, −49.31 to −30.45; Figure 4B and Supplementary Table S3).

### Effects of sacubitril/valsartan on left ventricular hypertrophy

**Figure 5:**
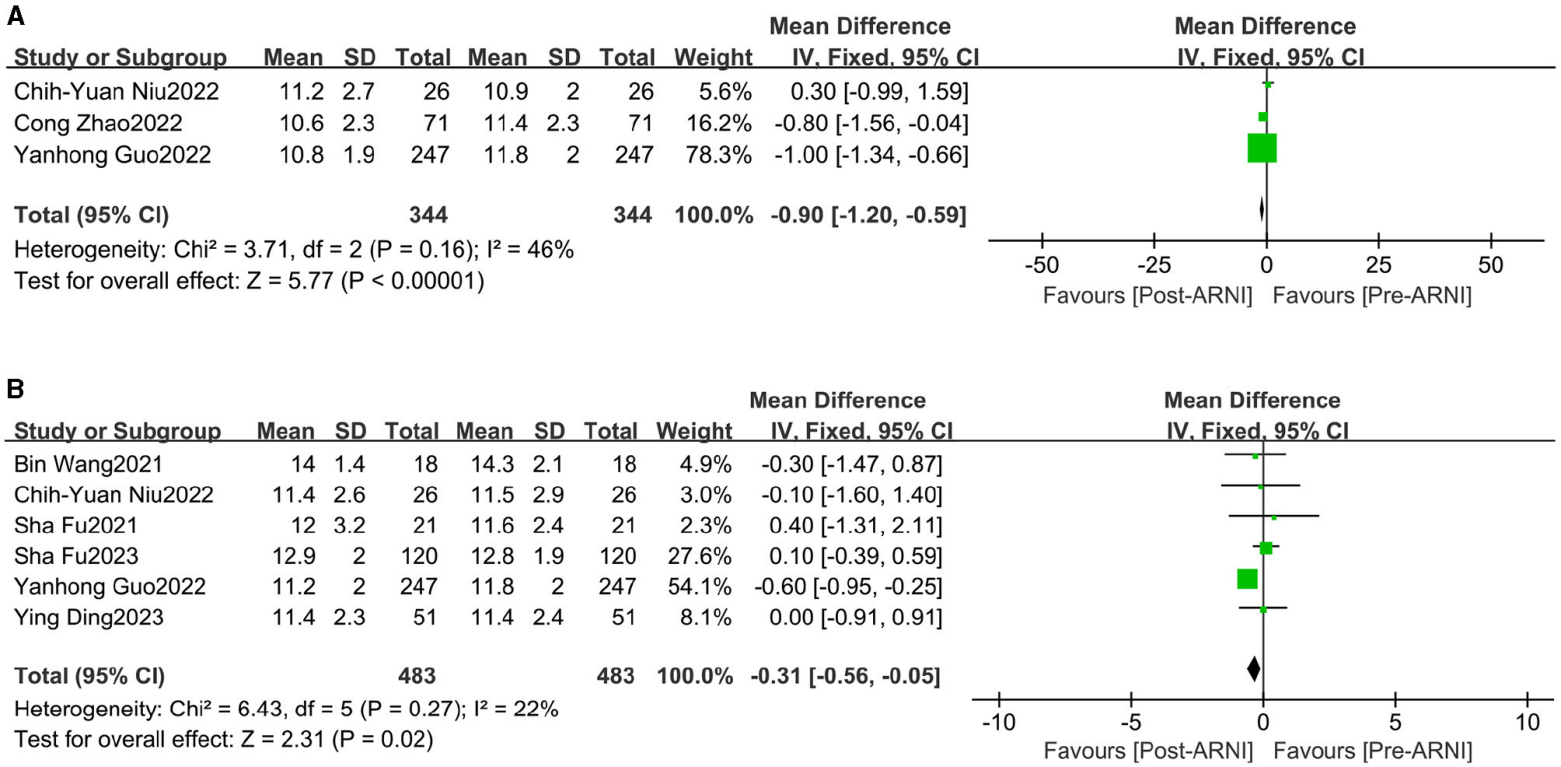
Forest plots showing changes in left ventricular posterior wall thickness (LVPWT) **(A)** and interventricular septum thickness in diastole (IVSd) **(B)**.

Changes in left ventricular posterior wall thickness (LVPWT) were observed in 344 patients and interventricular septum thickness in diastole (IVSD) in 483 patients across three (16, 23, 24) and six (16, 18, 20, 21, 24, 25) trials, respectively. Significant reductions were observed in LVPWT (MD, −0.90 mm; 95% CI, −1.20 to −0.59; Figure 5 and Supplementary Table S3) and IVSD (MD, −0.31 mm; 95% CI, −0.56 to 0.05 mm Hg; I^2^ = 67.9%; Figure 5 and Supplementary Table S3).

### Effects of sacubitril/valsartan on biomarkers for dialysis

**Figure 6:**
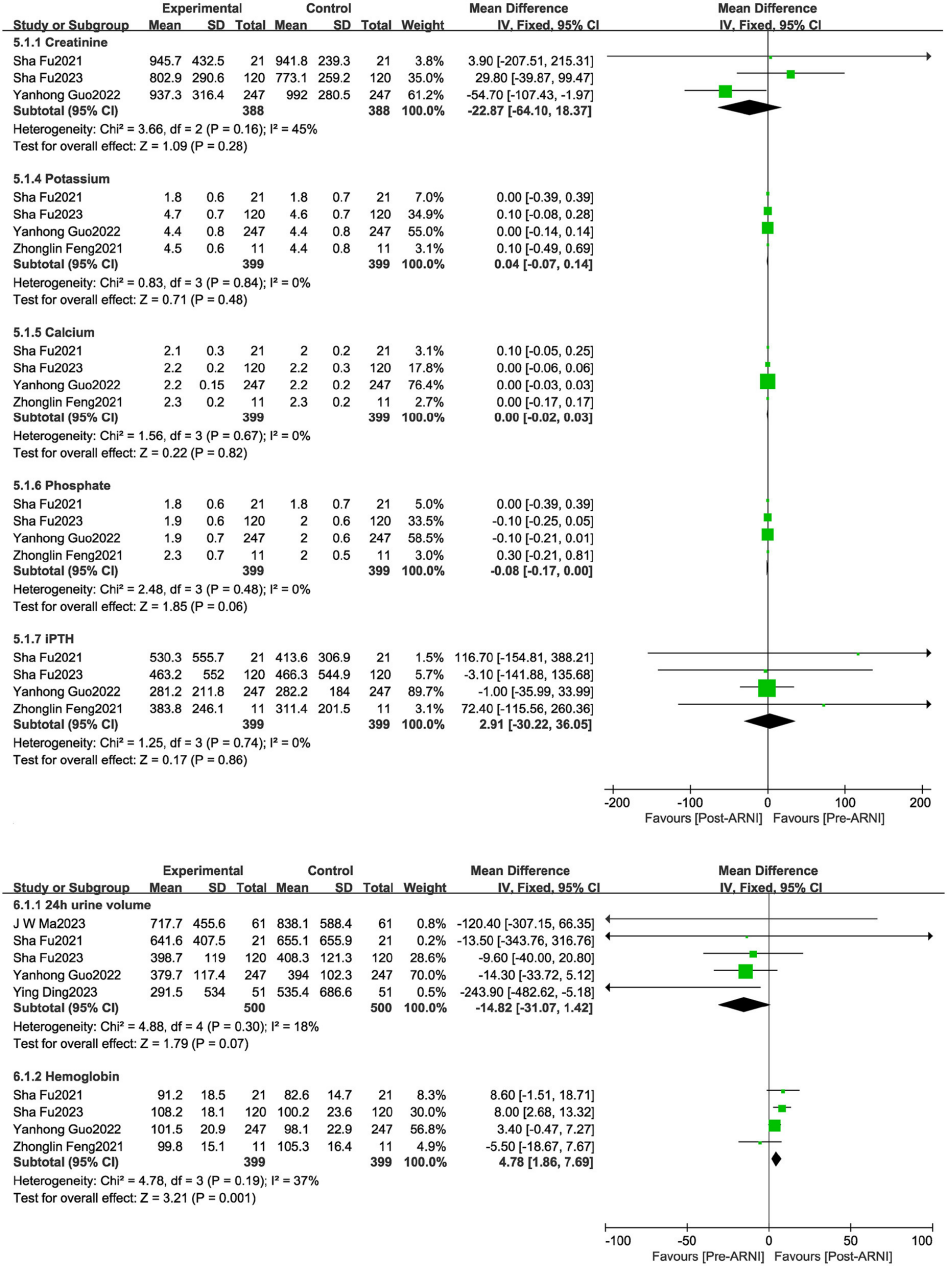
Forest plots showing changes in hemoglobin, potassium, creatinine, calcium, phosphorus, intact parathyroid hormone (PTH), and 24-h urine volume.

There were no significant improvements in creatinine, calcium, phosphorus, 24-h urine volume, or intact parathyroid hormone (iPTH). However, the pooled data from four studies (19, 21, 24, 25), involving 399 patients, showed increases in hemoglobin (MD, 4.78 g/L; 95% CI, 1.86 to 7.69 g/L; Figure 6).

### Effects of sacubitril/valsartan on LVEF and main indices compared with angiotensin-converting enzyme inhibitors/angiotensin receptor blockers

**Figure 7:**
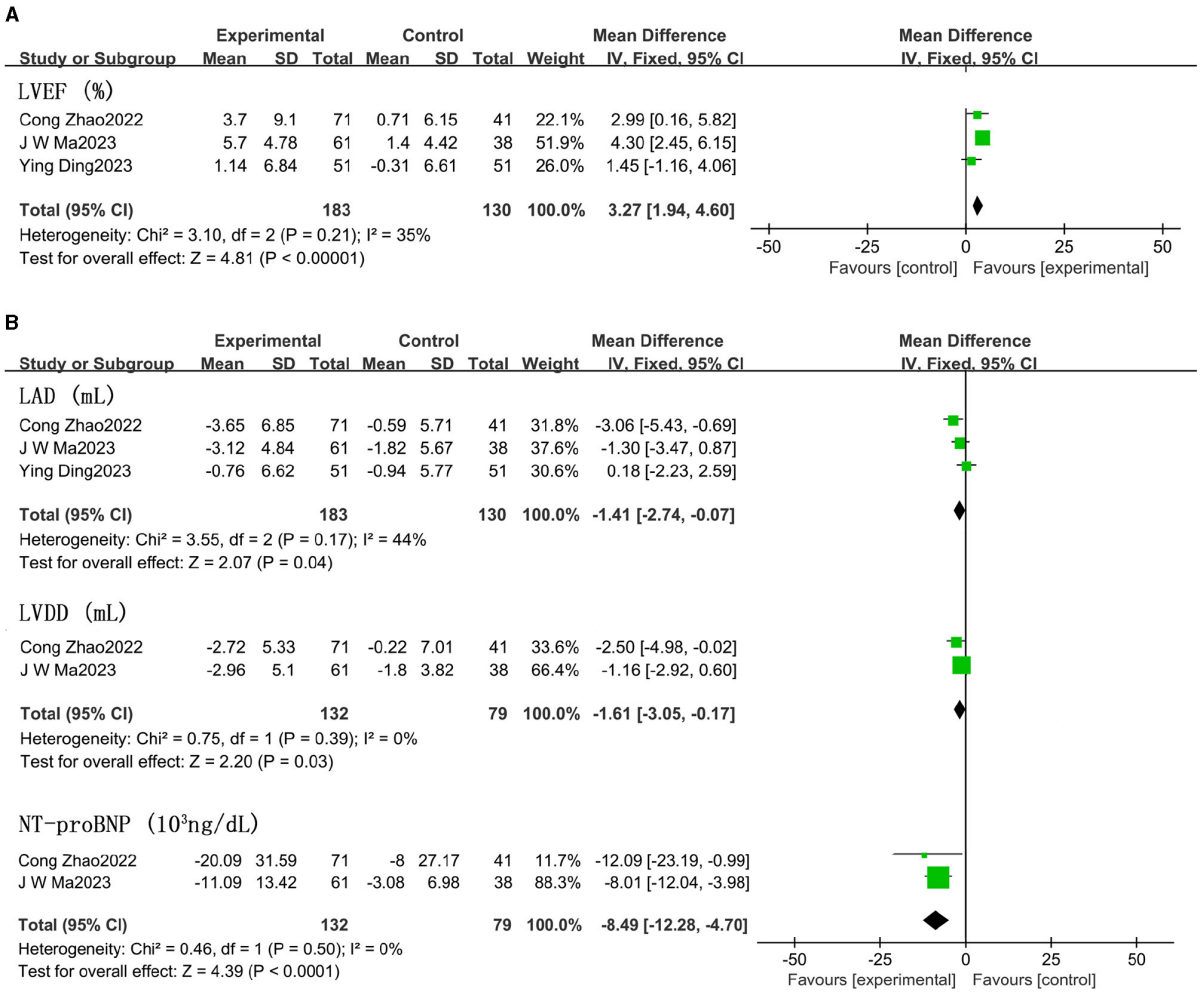
Effect of ARNI on LVEF and main indices (LVDD, LAD) **(A)** and NT-proBNP **(B)** compared with ACEIs/ARBs.

LVEF scores increased by 3.27% in patients treated with ARNI compared to those using ACEIs/ARBs (95% CI 1.94, 4.60; Figure 7A and Supplementary Table S4). Both left atrial dimension (LAD) (MD −1.41 mm, 95% CI −2.74, −0.07; Figure 7B and Supplementary Table S4) and left ventricular end-diastolic diameter (LVDD) significantly decreased (MD −1.61 mm, 95% CI −3.05, −0.17), and NT-proBNP showed a notable decline (MD −8.49 × 10^3^ ng/dL, 95% CI −12.28, −4.70; Figure 7B) in patients taking ARNI. However, there were no significant improvements in right ventricular diameter (RVD) or right atrial dimension (RAD) in patients treated with ARNI compared to those using ACEIs/ARBs (Supplementary Figure S2).

### Subgroup analyses

The results of subgroup analyses are presented in Table 2. In terms of blood pressure, the outcomes of the subgroup analysis based on six baseline characteristics were all statistically significant. An increase in LVEF was related to patients with a duration of dialysis ≥36 months and LVEF ≥50%. Similarly, it was observed that patients with a longer follow-up period were found to have better improvements in LVEF. However, no significant differences in LVEF changes were found in relation to other baseline characteristics. The subgroup analysis failed to provide a consistent explanation for the high heterogeneity (I^2^ = 91%) between studies concerning LVEF, although the I^2^ value decreased to 77.2% (Supplementary Figures S3, S4).

**Table 2:** Subgroup analysis of the effects of ARNI on blood pressure and LV indices by characteristics.

**Subgroup**	**No. of studies**	**SBP (mmHg)**	**DBP (mmHg)**	**NT–proBNP, 10^3^ pg/Ml**	**LVEF, %**	**LVESV, mL**
**Age, (years)**						
≥55	4	−13.50	−5.96	−5.31	3.8	−25.60
		(−17.79 −9.28)	(−8.87 −3.04)	(−7.29 −3.33)	(2.24 5.36)	(−44.37 −6.84)
		I^2^ = 72.7%	I^2^ = 0.0%	I^2^ = 0.0%	I^2^ = 90.7%	I^2^ = ^*^
		z = −6.23	z = −4.01	z = −5.26	z = 4.78	z = −2.67
		(*p =* 0.000)	(*p =* 0.000)	(*p =* 0.000)	(*p =* 0.000)	(*p =* 0.007)
<55	6	−15.73	−6.64	−11.17	1.38	−9.59
		(−18.54 −12.92)	(−8.72 −4.56)	(−13.51 −8.83)	(0.59 2.17)	(−13.39 −5.78)
		I^2^ = 77.2%	I^2^ = 30.1%	I^2^ = 76.5%	I^2^ = 91.9%	I^2^ = 28.0%
		z = −10.964	z = −6.26	z = −9.36	z = 3.43	z = −4.93
		(*p =* 0.000)	(*p =* 0.000)	(*p =* 0.000)	(*p =* 0.001)	(*p =* 0.000)
**Duration of dialysis (months)**						
≥36	4	−15.81	−5.59	−6.40	7.79	−12.33
		(−20.59 −11.03)	(−9.69 −1.49)	(−11.21 −1.59)	(5.51 10.07)	(−20.20 −4.46)
		I^2^ = 82.1%	I^2^ = 1.9%	I^2^ = 21.2%	I^2^ = 79.6%	I^2^ = 57.1%
		z = −6.48	z = −2.67	z = −2.61	z = 6.70	z = −3.07
		(*p =* 0.000)	(*p =* 0.008)	(*p =* 0.009)	(*p =* 0.000)	(*p =* 0.002)
<36	6	−14.12	−6.52	−11.65	1.2	−9.61
		(−17.39 −10.85)	(−8.79 −4.25)	(−14.04 −9.27)	(0.42 1.98)	(−13.85 −5.37)
		I^2^ = 75.4%	I^2^ = 49.9%	I^2^ = 77.3%	I^2^ = 93.1%	I^2^ = 64.0%
		z = −8.46	z = −5.62	z = −9.57	z = 3.02	z = −4.44
		(*p =* 0.000)	(*p =* 0.000)	(*p =* 0.000)	(*p =* 0.003)	(*p =* 0.000)
**Dialysis modality**						
HD	5	−14.85	−6.00	−6.97	0.71	NR
		(−17.43 −12.27)	(−7.85 −4.14)	(−8.81 −5.14)	(−0.12 1.54)	
		I^2^ = 73.6%	I^2^ = 0%	I^2^ = 87.4%	I^2^ = 91.3%	
		z = −11.29	z = −6.34	z = −7.45	z = 1.68	
		(*p =* 0.000)	(*p =* 0.000)	(*p =* 0.000)	(*p =* 0.094)	
PD	5	−17.73	−8.67	−9.42	4.31	NR
		(−23.60 −11.85)	(−13.00 −4.34)	(−12.08 −6.75)	(2.92 5.70)	
		I^2^ = 83.4	I^2^ = 62.7%	I^2^ = 14.2%	I^2^ = 77.6%	
		z = −5.92	z = −3.92	z = −6.92	z = 6.07	
		(*p =* 0.000)	(*p =* 0.000)	(*p =* 0.000)	(*p =* 0.000)	
**LVEF, %**						
≥50	7	−16.21	−6.81	−7.67	1.33	−9.60
		(−18.66 −13.76)	(−8.58 −5.04)	(−9.20 −6.14)	(0.61 2.06)	(−13.39 −5.78)
		I^2^ = 72.5%	I^2^ = 13.9%	I^2^ = 83.3%	I^2^ = 89.9%	I^2^ = 28%
		z = −12.98	z = −7.53	z = −9.81	z = 3.61	z = −4.93
		(*p =* 0.000)	(*p =* 0.000)	(*p =* 0.000)	(*p =* 0.000)	(*p =* 0.000)
<50	3	−2.04	−2.24	−10.90	11.3	−25.60
		(−10.28 6.19)	(−7.97 3.50)	(−20.10 −1.71)	(8.29 14.32)	(−44.37 −6.84)
		I^2^ = 0.0%	I^2^ = 0.0%	I^2^ = ^*^	I^2^ = 21.9%	I^2^ = ^*^
		z = −0.49	z = −0.77	z = −2.32	z = 7.35	z = −2.67
		(*p =* 0.627)	(*p =* 0.445)	(*p =* 0.020)	(*p =* 0.000)	(*p =* 0.007)
**Follow–up duration (months)**						
≥6	6	−14.90	−6.54	−6.80	4.27	−25.60
		(−18.31 −11.49)	(−8.93 −4.14)	(−8.44 −5.16)	(3.12 5.42)	(−44.37 −6.84)
		I^2^ = 70.8%	I^2^ = 35.0%	I^2^ = 59.3%	I^2^ = 82.3%	I^2^ = ^*^
		z = −8.56	z = −5.35	z = −8.122	z = 7.300	z = −2.67
		(*p =* 0.000)	(*p =* 0.000)	(*p =* 0.000)	(*p =* 0.000)	(*p =* 0.007)
<6	4	−15.21	−6.28	−13.10	0.43	−9.59
		(−18.44 −11.98)	(−8.68 −3.89)	(−16.97 −9.22)	(−0.47 1.32)	(−13.39 −5.78)
		I^2^ = 83.1%	I^2^ = 0.0%	I^2^ = 88.8%	I^2^ = 93.5%	I^2^ = 28.0%
		z = −9.222	z = −5.14	z = −6.62	z = 0.94	z = −4.93
		(*p =* 0.000)	(*p =* 0.000)	(*p =* 0.000)	(*p =* 0.350)	(*p =* 0.000)
**Sample size**						
≥50	5	−15.32	−6.75	−8.03	1.26	−9.61
		(−18.06 −12.57)	(−8.63 −4.87)	(−9.67 −6.39)	(0.52 2.00)	(−13.85 −5.37)
		I^2^ = 72.6%	I^2^ = 20.1%	I^2^ = 88.3%	I^2^ = 93.1%	I^2^ = 64.0%
		z = −10.95	z = −7.04	z = −9.598	z = 3.33	z = −4.44
		(*p =* 0.000)	(*p =* 0.000)	(*p =* 0.000)	(*p =* 0.001)	(*p =* 0.000)
<50	5	−14.37	−4.94	−6.23	7.3	−12.33
		(−18.91 −9.83)	(−8.83 −1.04)	(−10.12 −2.33)	(5.09 9.50)	(−20.20 −4.46)
		I^2^ = 80.3%	I^2^ = 1.7%	I^2^ = 0.0%	I^2^ = 77.2%	I^2^ = 57.1%
		z = −6.21	z = −2.48	z = −3.13	z = 6.48	z = −3.07
		(*p =* 0.000)	(*p =* 0.013)	(*p =* 0.002)	(*p =* 0.000)	(*p =* 0.002)
**Study design**						
Prospective	3	−17.33	−6.59	−4.88	−3.69	−12.33
		(−20.82 −13.84)	(−9.25 −3.94)	(−6.87 −2.88)	(−5.50 −1.88)	(−20.20 −4.46)
		I^2^ = 78.5%	I^2^ = 11.9%	I^2^ = 0.0%	I^2^ = 89.3%	I^2^ = 57.1%
		z = −9.73	z = −4.87	z = −4.78	z = −3.99	z = −3.07
		(*p =* 0.000)	(*p =* 0.000)	(*p =* 0.000)	(*p =* 0.000)	(*p =* 0.002)
Retrospective	7	−13.20	−6.28	−11.60	−1.56	−9.61
		(−16.37 −10.03)	(−8.48 −4.09)	(−13.91 −9.30)	(−2.32 −0.79)	(−13.85 −5.37)
		I^2^ = 69.9%	I^2^ = 19.1%	I^2^ = 71.6%	I^2^ = 92.3%	I^2^ = 64.0%
		z = −8.17	z = −5.61	z = −9.85	z = −3.99	z = −4.44
		(*p =* 0.000)	(*p =* 0.000)	(*p =* 0.000)	(*p =* 0.000)	(*p =* 0.000)
**Subgroup**	**No. of studies**	**LVEDV, mL**	**LVDd, mm**	**E/e' ratio**	**LAD, mm**	**IVSD, mm**
**Age (years)**						
≥55	4	−16.10	0.99	0.47	−0.31	0.08
		(−37.86 5.66)	(−0.48 2.45)	(−0.26 1.19)	(−1.68 1.07)	(−0.39 0.55)
		I^2^ = ^*^	I^2^ = 59.4%	I^2^ = 92.2%	I^2^ = 44.8%	I^2^ = 0.0%
		z = −1.45	z = 1.32	z = 1.26	z = −0.44	z = 0.34
		(*p =* 0.147)	(*p =* 0.187)	(*p =* 0.207)	(*p =* 0.661)	(*p =* 0.732)
<55	6	−12.51	−2.76	−1.53	−0.35	−0.50
		(−18.92 −6.11)	(−3.62 −1.90)	(−2.09 −0.97)	(−1.18 0.48)	(−0.82 −0.18)
		I^2^ = 4.5%	I^2^ = 0.0%	I^2^ = 0.0%	I^2^ = 66.0%	I^2^ = 0.0%
		z = −3.83	z = −6.30	z = −5.37	z = −0.83	z = −3.08
		(*p =* 0.000)	(*p =* 0.000)	(*p =* 0.000)	(*p =* 0.407)	(*p =* 0.002)
**Duration of dialysis (months)**						
≥36 月	4	−12.68	−3.10	−4.74	−3.20	−0.21
		(−26.07 0.71)	(−5.84 −0.37)	(−8.38 −1.10)	(−7.63 1.23)	(−1.30 0.88)
		I^2^ = 0.0%	I^2^ = ^*^	I^2^ = 83.8%	I^2^ = ^*^	I^2^ = 0.0%
		z = −1.86	z = −2.22	z = −2.56	z = −1.42	z = −0.37
		(*p =* 0.063)	(*p =* 0.026)	(*p =* 0.011)	(*p =* 0.16)	(*p =* 0.710)
<36 月	6	−12.83	−2.50	−1.52	−0.35	−0.51
		(−19.75 −5.91)	(−3.35 −1.65)	(−2.08 −0.96)	(−1.18 0.48)	(−0.83 −0.18)
		I^2^ = 50.9%	I^2^ = 0.0%	I^2^ = 38.8%	I^2^ = 66.0%	I^2^ = 40.7%
		z = −3.64	z = −5.74	z = −5.29	z = −0.83	z = −3.07
		(*p =* 0.000)	(*p =* 0.000)	(*p =* 0.000)	(*p =* 0.407)	(*p =* 0.002)
**Dialysis modality**						
HD	5	NR	−1.64	−0.73	−0.40	−0.36
			(−2.50 −0.78)	(−1.17 −0.28)	(−1.23 0.43)	(−0.64 −0.08)
			I^2^ = 84.0%	I^2^ = 90.2%	I^2^ = 76.0%	I^2^ = 60.9%
			z = −3.75	z = −3.21	z = 0.22	z = −2.51
			(*p =* 0.000)	(*p =* 0.001)	(*p =* 0.823)	(*p =* 0.012)
PD	5	NR	−2.25	−8.60	0.17	0
			(−3.71 −0.79)	(−19.47 2.27)	(−1.29 1.627)	(−0.83 0.83)
			I^2^ = 52.1%	I^2^ = ^*^	I^2^ = 0.0%	I^2^ = ^*^
			z = −3.03	z = −1.55	z = 0.22	z = 0.00
			(*p =* 0.002)	(*p =* 0.121)	(*p =* 0.823)	(*p =* 1.000)
**LVEF, %**						
≥50	7	−12.51	NR	−0.73	−0.26	−0.33
		(−18.92 −6.11)		(−1.17 −0.28)	(−0.99 0.46)	(−0.60 −0.05)
		I^2^ = 4.5%		I^2^ = 90.9%	I^2^ = 55.5%	I^2^ = 65.3%
		z = −3.83		z = −3.19	z = −0.72	z = −2.35
		(*p =* 0.000)		(*p =* 0.001)	(*p =* 0.473)	(*p =* 0.019)
<50	3	−16.10	NR	−4.74	−3.20	−0.21
		(−37.86 5.66)		(−8.38 −1.10)	(−7.63 1.23)	(−1.30 0.88)
		I^2^ = ^*^		I^2^ = 83.8%	I^2^ = ^*^	I^2^ = 0.0%
		z = −1.45		z = −2.56	z = −1.42	z = −0.372
		(*p =* 0.147)		(*p =* 0.011)	(*p =* 0.157)	(*p =* 0.710)
**Follow–up duration (months)**						
≥6月	6	−16.10	−0.63	0.49	0.08	0.05
		(−37.86 5.66)	(−1.76 0.50)	(−0.23 1.20)	(−0.94 1.11)	(−0.36 0.46)
		I^2^ = ^*^	I^2^ = 85.7%	I^2^ = 51.3%	I^2^ = 0.0%	I^2^ = 0.0%
		z = −1.45	z = −1.09	z = 1.33	z = 0.16	z = 0.22
		(*p =* 0.147)	(*p =* 0.278)	(*p =* 0.182)	(*p =* 0.875)	(*p =* 0.825)
<6 月	4	−12.51	−2.69	−1.57	−0.7	−0.58
		(−18.92 −6.11)	(−3.67 −1.71)	(−2.13 −1.01)	(−1.71 0.26)	(−0.92 −0.23)
		I^2^ = 4.5%	I^2^ = 0.0%	I^2^ = 89.0%	I^2^ = 78.2%	I^2^ = 0.0%
		z = −3.83	z = −5.37	z = −5.47	z = −1.45	z = −3.28
		(*p =* 0.000)	(*p =* 0.000)	(*p =* 0.000)	(*p =* 0.147)	(*p =* 0.001)
**Sample size**						
≥50	5	−12.83	−1.70	−0.73	−0.25	−0.33
		(−19.75 −5.91)	(−2.47 −0.93)	(−1.17 −0.28)	(−0.98 0.48)	(−0.60 −0.05)
		I^2^ = 50.9%	I^2^ = 80.4%	I^2^ = 90.9%	I^2^ = 66.5%	I^2^ = 65.3%
		z = −3.64	z = −4.32	z = −3.19	z = −0.67	z = −2.35
		(*p =* 0.000)	(*p =* 0.000)	(*p =* 0.001)	(*p =* 0.505)	(*p =* 0.019)
<50	5	−12.68	−3.10	−4.74	−1.82	−0.21
		(−26.07 0.71)	(−5.84 −0.37)	(−8.38 −1.10)	(−4.79 1.15)	(−1.30 0.88)
		I^2^ = 0.0%	I^2^ = ^*^	I^2^ = 83.8%	I^2^ = 0.0%	I^2^ = 0.0%
		z = −1.86	z = −2.22	z = −2.56	z = −1.20	z = −0.37
		(*p =* 0.063)	(*p =* 0.026)	(*p =* 0.011)	(*p =* 0.229)	(*p =* 0.710)
**Study design**						
Prospectively	3	0.34	−2.81	0.7	0.03	0.34
		(−1.16 1.83)	(−5.81 0.18)	(−0.71 2.11)	(−0.41 0.46)	(−1.16 1.83)
		I^2^ = 88.4%	I^2^ = 22.0%	I^2^ = 73.7%	I^2^ = 0.0%	I^2^ = 88.4%
		z = 0.45	z = −1.84	z = 0.97	z = 0.12	z = 0.45
		(*p =* 0.657)	(*p =* 0.066)	(*p =* 0.330)	(*p =* 0.902)	(*p =* 0.657)
Retrospectively	7	−2.50	−1.51	−2.73	−0.49	−2.50
		(−3.35 −1.65)	(−2.07 −0.94)	(−3.55 −1.91)	(−0.81 −0.17)	(−3.35 −1.65)
		I^2^ = 0.0%	I^2^ = 0.0%	I^2^ = 57.7%	I^2^ = 20.7%	I^2^ = 0.0%
		z = −5.74	z = −5.24	z = −6.52	z = −2.97	z = −5.74
		(*p =* 0.000)	(*p =* 0.000)	(*p =* 0.000)	(*p =* 0.003)	(*p =* 0.000)

Results at 3- to 12-month follow-up were used unless otherwise stated. Mean differences are pooled meta-analysis estimates with 95% CIs. I^2^ values are reported as a measure of heterogeneity. Z scores with associated *p*-values are reported as tests of overall effect. ACEI indicates angiotensin-converting enzyme inhibitor; ARB, angiotensin receptor blocker; ARNI, angiotensin receptor neprilysin inhibitor; SBP, systolic blood pressure; DBP, diastolic blood pressure; NT-proBNP, N-terminal pro-B-type natriuretic peptide; LVEF, left ventricular ejection fraction; LVESV, left ventricular end-systolic volume; LVEDV, left ventricular end-diastolic volume; LVDd, left ventricular end-diastolic diameter; LVDs, left ventricular end-systolic diameter; E/e' ratio, the ratio of early mitral inflow velocity to mitral annular early diastolic velocity; peak TR Vel, peak tricuspid regurgitation velocity; LAD, left atrial dimension; LVPWT, left ventricular posterior wall thickness; IVSd, diastolic interventricular septum thickness. ^*^Data were available in only one study.

A subgroup analysis according to the follow-up period showed significant effects of ARNI on blood pressure and left ventricular functional capacity at 6 months, with improvements increasing over time. In the analyses of LV function indices, age, baseline LVEF, dialysis modality, duration of dialysis, and sample size were not associated with significant improvements (Table 2).

Subgroup analyses based on study design indicated that both blood pressure and ejection fraction showed considerable improvement in both prospective and retrospective studies (Supplementary Figures S5, S6).

### Safety of sacubitril/valsartan

**Figure 8:**
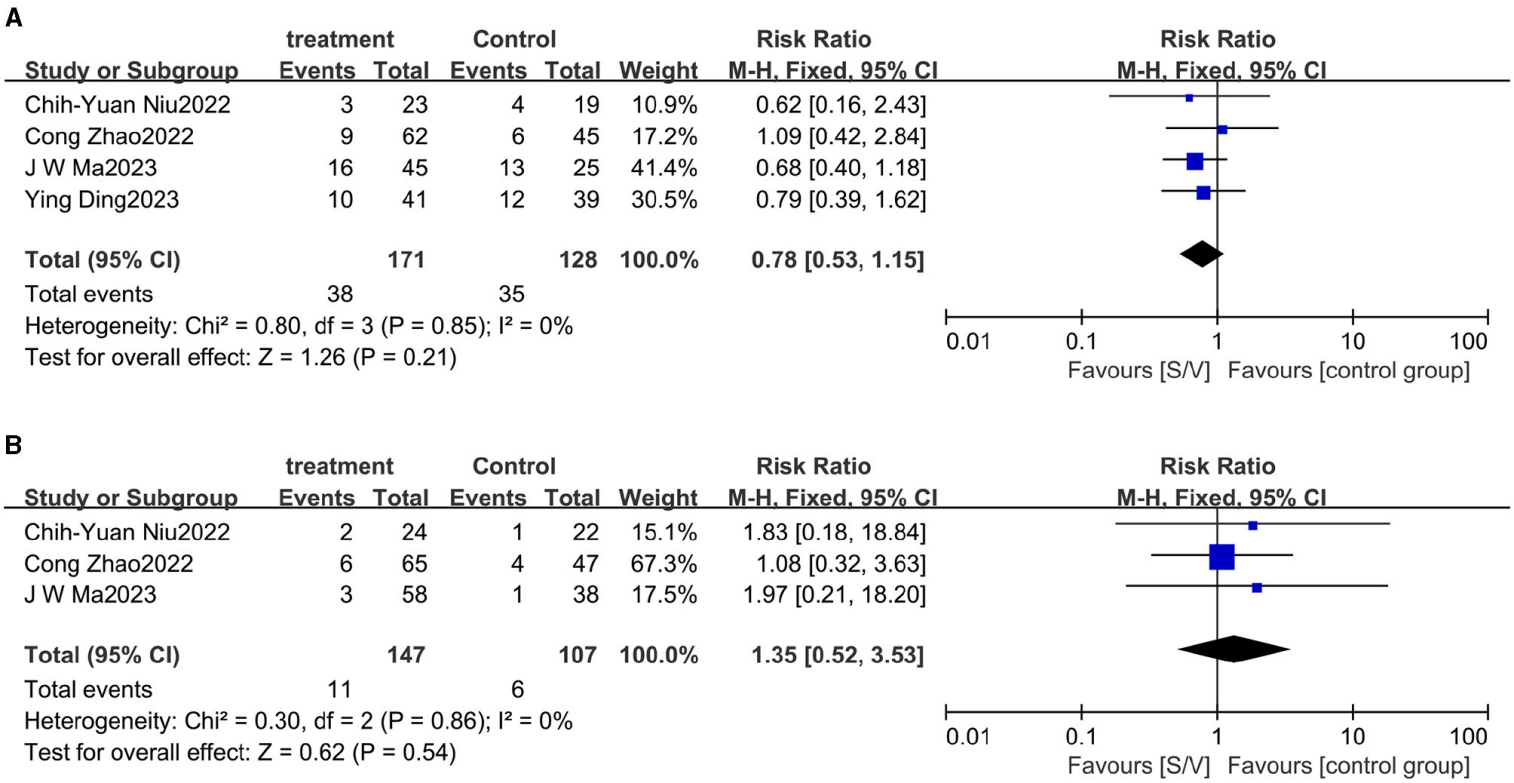
Forest plots of major adverse events. **(A)** Hyperkalemia. **(B)** Hypotension.

Four studies reported on hyperkalemia rates. In the ARNI group, 38 instances of hyperkalemia (21.0%) were noted, compared to 35 cases (27.3%) in the control group. The difference in hyperkalemia rates between the groups was not statistically significant, with a risk ratio (RR) of 0.78 (95% CI 0.53 to 1.15, *p* = 0.85) (Figure 8A). Similarly, three studies reported on hypotension occurrences. There was no significant difference in the incidence of hypotension between the groups, with an RR of 1.35 (95% CI 0.52 to 3.53, *p* = 0.86) (Figure 8B).

### Publication bias and sensitivity analysis

**Figure 9:**
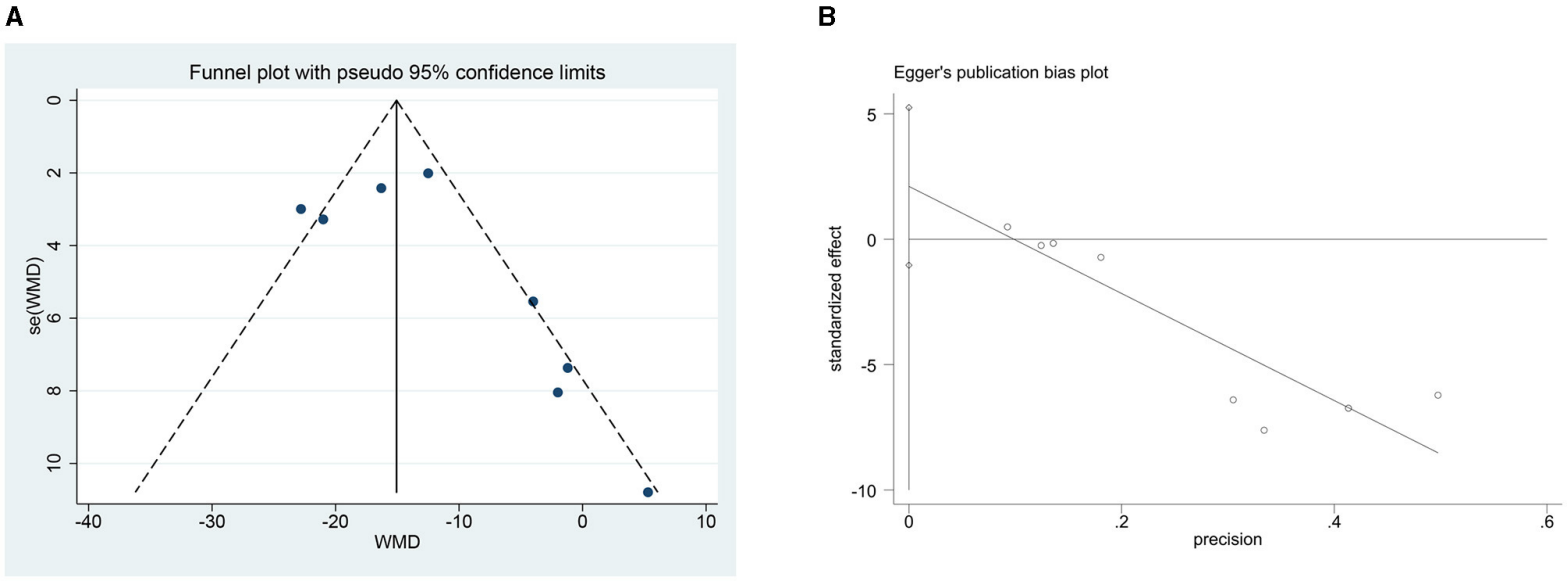
**(A)** Funnel plot of the SBP change. **(B)** Publication bias test for the SBP change.

We plotted the funnel plots along with Egger's test to evaluate the SBP's publication bias in STATA 15.1 software. The funnel plot showed that these studies were approximately symmetrically distributed on both sides of the regression line. Additionally, the results from Egger's test showed no significant publication bias for the SBP (*p* = 0.152) (Figure 9).

We also performed sensitivity analyses for these 10 studies by excluding each article to validate the stability of this meta-analysis, which revealed that there was no statistical effect on the pooled data when any of the articles were eliminated except for Yanhong Guo's work from 2022. However, the findings of this study had to be interpreted with caution when explaining the prognostic results of LVEF (Supplementary Figure S7).

### Correlation analyses

Analyses of LVEF and other left ventricular (LV) function indices, which followed normal distributions, were conducted using Pearson's correlation coefficient to explore potential relationships. No significant correlation was found between LVEF improvements and reductions in other LV indices. The results were as follows: LVEF and systolic blood pressure (SBP) (*r* = 0.449, *p* = 0.264), LVEF and diastolic blood pressure (DBP) (*r* = 0.085, *p* = 0.841), LVEF and left ventricular end-systolic volume (LVESV) (*r* =−0.983, *p* = 0.017), LVEF and left ventricular end-diastolic volume (LVEDV) (*r* =−0.498, *p* = 0.502), LVEF and left ventricular end-diastolic diameter (LVDd) (*r* =−0.346, *p* = 0.502), LVEF and E/e' ratio (*r* =−0.735, *p* = 0.265), LVEF and left atrial dimension (LAD) (*r* =−0.457, *p* = 0.303), and LVEF and interventricular septum thickness in diastole (IVSD) (*r* =−0.008, *p* = 0.987) (Supplementary Figure S8). A significant negative correlation was only observed between LVEF and LVESV, indicating a potential relationship between improved ejection fraction and reduced end-systolic volume.

## Discussion

This study presents the first meta-analysis evaluating the effects of the angiotensin receptor neprilysin inhibitor (ARNI) on blood pressure, left ventricular function, and biomarkers in dialysis patients. The results demonstrate that ARNI significantly ameliorates recalcitrant hypertension and facilitates reverse ventricular remodeling in this population. Additionally, ARNI showed superior efficacy in improving left ventricular function compared to ACEIs or ARBs. Sacubitril/valsartan was also found to be well tolerated in patients with end-stage renal disease (ESRD).

Recent trials have established the efficacy of ARNI in managing blood pressure in patients with or without chronic kidney disease (CKD). In 2018, studies confirmed the effectiveness of sacubitril/valsartan (SV) monotherapy in patients with uncontrolled hypertension previously treated with olmesartan (26). Notably, SV has been shown to be superior in lowering blood pressure compared to renin-angiotensin-aldosterone system (RAAS) inhibitors (12, 27). This aligns with the findings of our study, which identifies hypertension as a crucial modifiable factor in the development of heart failure (28) and acknowledges that prolonged pressure overload can induce cardiac remodeling (29). Our analysis revealed significant reductions in both systolic and diastolic blood pressure with ARNI treatment compared to controls. This finding suggests that controlling blood pressure and reducing volumetric load may contribute to improved cardiac function and reverse ventricular remodeling.

Furthermore, we observed a significant reduction in N-terminal proB-type natriuretic peptide (NT-proBNP), not only through its effects on humoral homeostasis but also by modulating left ventricular load through vascular tone regulation. This contributes to decreased systemic vascular resistance, which supports reverse cardiac remodeling (30). NT-pro-BNP is a useful biomarker for heart failure (HF) in the general population. The PARAMOUNT phase II trial identified NT-pro-BNP as the primary outcome and demonstrated that 12-week treatment with sacubitril/valsartan reduced NT-pro-BNP levels by 23%, indicating a potential clinical benefit (31). However, while NT-pro-BNP is a surrogate marker for HF in early-stage kidney disease, its reliability diminishes in advanced kidney disease (32, 33). Patients with advanced kidney disease often exhibit elevated NT-pro-BNP levels due to their primary metabolism through the kidneys. Consequently, in dialysis patients, NT-pro-BNP levels can reach extremely high values and are generally unreliable, providing no conclusive evidence for diagnosing HF, whether to confirm or exclude it.

In previous studies, ARNI significantly impacted LV function in heart failure patients, including those who failed to reach the target dose of either an ACE inhibitor or an ARB. Both ACE inhibitors and ARBs are well-recognized for improving prognosis in patients with heart failure and myocardial infarction, demonstrating a beneficial effect in reducing cardiovascular mortality and reversing myocardial remodeling (34–38). Consequently, it is plausible that an ARNI, combining the effects of an ARB and a neprilysin inhibitor, would favorably influence LV cardiac function. Our data further extend these benefits to patients with ESKD, with Sacubitril/Valsartan significantly enhancing LV systolic and diastolic functions in these patients. A recent study using strain echocardiography showed that a 6-month treatment with Sacubitril/Valsartan could improve LV global longitudinal strain, twist, and apical and basal rotations, thereby effectively alleviating myocardial wall tension (39).

This meta-analysis generally compares patients treated with ACEIs or ARBs and emphasizes that ARNI significantly improves left ventricular function. However, our study found no significant difference in structural changes of the right ventricle in dialysis patients treated with ARNIs compared to those treated with ACEIs/ARBs, suggesting that ARNIs may not provide a substantial advantage in improving the right ventricular structure.

The current subgroup analysis demonstrated robust results for significant improvement in left ventricular function, regardless of dialysis modality, duration, or age. Interestingly, improvement in left ventricular function was associated with ejection fraction. It was observed that dialysis patients without preserved ejection fraction achieved better improvements, including blood pressure reduction, than those with preserved ejection fraction. Improvement in left ventricular function was significant in long-term dialysis patients, who may suffer from severe vascular damage and ventricular remodeling due to prolonged hypertension and volume loading. ARNI improves left ventricular function by reversing cardiac remodeling and mitigating recalcitrant hypertension through reduced peripheral resistance. These findings should be interpreted with caution due to the loss of statistical power and indirect comparisons. Further studies are required to directly compare the improvement in left ventricular function after ARNI treatment in patients with varying durations of dialysis.

This study's large and small sample sizes highlighted significant improvements in left ventricular function, indicating that both sampling strategies possess strong scientific validity. This evidence supports the broad applicability of the findings, with no significant differences observed between different dialysis modalities. The study highlights that both hemodialysis and peritoneal dialysis patients benefit from the use of ARNI, which inhibits cardiac remodeling and controls blood pressure effectively. However, further prospective studies are needed to confirm these results.

Subgroup analyses based on follow-up duration indicated that ARNI can significantly improve left ventricular function as early as 6 months, with benefits increasing over time. This early effect is likely due to the high levels of NT-proBNP and severe fluid retention common in dialysis patients, where short-term use of ARNI proves particularly effective. The results suggest that the long-term benefits of ARNI, particularly in terms of sustained blood pressure improvement and cardiovascular prognosis, are significant. Therefore, early initiation of ARNI in eligible patients could be advantageous. Stratified comparisons by baseline characteristics revealed no significant differences or heterogeneity between studies for the majority of indicators, further validating the reliability of this meta-analysis.

We observed a linear relationship between left ventricular ejection fraction (LVEF) and left ventricular end-diastolic volume (LVEDV), marked by a high correlation coefficient. Improvements in LVEF correlated with greater reductions in LVEDV. This relationship is likely because dialysis patients with diminished ventricular function and abnormally enlarged ventricles may not exhibit a significant difference in stroke volume compared to normal controls but may have an increased LVEDV, resulting in a significantly lower ejection fraction. The role of ARNI in reversing ventricular remodeling contributes to decreased LV end-diastolic volume, ultimately enhancing ejection fraction. However, correlations between LVEF and other indices of left ventricular function were not determined. Given the small sample size, these findings should be interpreted with caution, and additional studies are needed to confirm the effects of ARNI on isolated LVEF improvements, independent of overall LV remodeling.

Our findings indicate that concerns regarding the occurrence of hyperkalemia and hypotension should not deter the use of ARNI in dialysis patients. *Traditionally*, hyperkalemia has been a significant issue when inhibiting the renal angiotensin-aldosterone system, especially in patients with renal impairment. However, the addition of neprilysin inhibition does not appear to elevate the risk of hyperkalemia in patients already receiving renin-angiotensin-aldosterone system inhibitors. Evidence from a meta-analysis in patients with heart failure with reduced ejection fraction (HFrEF) indicated that the incidence of hyperkalemia was lower in patients treated with ARNI compared to those treated with ACEIs (40). In our meta-analysis, no increase in the rate of hyperkalemia was observed following the initiation of ARNI therapy. Based on these findings, we conclude that ARNI is well tolerated in dialysis patients.

## Strengths and limitations

### Strengths

This meta-analysis is the first to compare the effects of ARNI on blood pressure and cardiac function in dialysis patients, providing evidence for the effectiveness of ARNI treatment. We conducted subgroup analyses based on baseline characteristics to address potential heterogeneity. The low level of heterogeneity among the data suggests that the observations are robust and likely to be valid across various settings.

### Limitations

However, this meta-analysis is not without limitations. First, the primary limitation is that the included studies are observational, which limits the ability to infer causality. While only four of the included studies had control groups, many patients were on stable ACEI/ARB therapy before starting ARNI, and we noted gradual improvements after switching to ARNI. This indicates potential benefits, but randomized controlled trials are needed to establish a direct causal relationship. Second, as is the case with many meta-analyses, the included studies generally had small sample sizes. This may affect the power of the analysis to detect smaller effects and may increase the potential for statistical error. Third, due to the limited number of studies examining the effects of ARNI on the cardiac system, specifically in dialysis patients, the studies included were relatively concentrated in terms of year and geographic region of publication. This concentration may introduce bias related to specific population characteristics or healthcare practices, limiting the generalizability of the findings. In conclusion, while this meta-analysis provides valuable insights into the potential benefits of ARNI for dialysis patients, the results should be interpreted with caution.

## Conclusion

This meta-analysis demonstrates that sacubitril/valsartan (SV) significantly reduces blood pressure in dialysis patients. It confirms its beneficial effects on left ventricular (LV) function, which may be largely attributed to its capacity to reverse ventricular remodeling. These effects appear to be particularly pronounced in patients on long-term dialysis. Notably, S/V can produce significant therapeutic improvements in LV function within just 6 months, with benefits increasing over time. Therefore, eligible patients should initiate S/V therapy as early as possible.

Our findings also indicate that S/V maintains a relatively favorable safety profile for dialysis patients. However, additional prospective studies are required to more thoroughly determine the efficacy and safety of S/V in this patient population. These studies should aim to explore the long-term effects of ARNI on dialysis patients, thereby enhancing physicians' capacity to make early prognostic assessments and tailor treatment strategies accordingly.

## Data Availability

The original contributions presented in the study are included in the article/Supplementary material, further inquiries can be directed to the corresponding author.
