# Supplementary material for: Safety and efficacy of angiotensin receptor neprilysin inhibitor in improving cardiac function and blood pressure in dialysis patients
Source: Front Med (Lausanne). 2024 Sep 5;11:1421085. doi: 10.3389/fmed.2024.1421085 (PMC11410709; doi:10.3389/fmed.2024.1421085)
Supplement: Supplementary file 1 [file Table_1.docx]

**Supplemental Material**

**Data S1.**

**PubMed search strategy:**

(((((((("Renal Dialysis"[Mesh]) OR (((((((((Dialyses, Renal) OR (Renal Dialyses)) OR (Dialysis, Renal)) OR (Hemodialysis)) OR (Hemodialyses)) OR (Dialysis, Extracorporeal)) OR (Dialyses, Extracorporeal)) OR (Extracorporeal Dialyses)) OR (Extracorporeal Dialysis))) OR ("Hemofiltration"[Mesh])) OR (((((((Hemofiltrations) OR (Venovenous Hemofiltration)) OR (Hemofiltration, Venovenous)) OR (Venovenous Hemofiltrations)) OR (Arteriovenous Hemofiltration)) OR (Arteriovenous Hemofiltrations)) OR (Hemofiltration, Arteriovenous))) OR ("Kidney Failure, Chronic"[Mesh])) OR (( ((((((((((((((End-Stage Kidney Disease) OR (Disease, End-Stage Kidney)) OR (End Stage Kidney Disease)) OR (Kidney Disease, End-Stage)) OR (Chronic Kidney Failure)) OR (End-Stage Renal Disease)) OR (Disease, End-Stage Renal)) OR (End Stage Renal Disease)) OR (Renal Disease, End-Stage)) OR (Renal Disease, End Stage)) OR (Renal Failure, End-Stage)) OR (End-Stage Renal Failure)) OR (Renal Failure, End Stage)) OR (Renal Failure, Chronic)) OR (Chronic Renal Failure)) OR (ESRD))) OR ("Peritoneal Dialysis, Continuous Ambulatory"[Mesh])) OR ((CAPD) OR (Continuous Ambulatory Peritoneal Dialysis))) AND (("sacubitril and valsartan sodium hydrate drug combination" [Supplementary Concept]) OR ((((((((((((sacubitril valsartan sodium hydrate) OR (sacubitril-valsartan sodium hydrate drug combination)) OR (sacubitril and valsartan drug combination)) OR (sacubitril valsartan drug combination)) OR (sacubitril-valsartan)) OR (sacubitril and valsartan sodium anhydrous drug combination)) OR (sacubitril valsartan sodium anhydrous)) OR (sacubitril-valsartan sodium anhydrous drug combination)) OR (LCZ 696)) OR (LCZ696)) OR (LCZ-696)) OR (Entresto)))

Total 294

Embase search strategy: 1.'renal dialysis':ab,ti OR 'dialyses, renal':ab,ti OR 'renal dialyses':ab,ti OR 'dialysis, renal':ab,ti OR hemodialysis:ab,ti OR hemodialyseshemodialysis:ab,ti OR 'dialysis, extracorporeal':ab,ti OR 'dialyses, extracorporeal':ab,ti OR 'extracorporeal dialyses':ab,ti OR 'extracorporeal dialysis':ab,ti OR hemofiltration:ab,ti OR hemofiltrations:ab,ti OR 'venovenous hemofiltration':ab,ti OR 'hemofiltration, venovenous':ab,ti OR 'venovenous hemofiltrations':ab,ti OR 'arteriovenous hemofiltration':ab,ti OR 'arteriovenous hemofiltrations':ab,ti OR 'hemofiltration, arteriovenous':ab,ti OR 'kidney failure, chronic':ab,ti OR 'end-stage kidney disease':ab,ti OR 'disease, end-stage kidney':ab,ti OR 'end stage kidney disease':ab,ti OR 'kidney disease, end-stage':ab,ti OR 'chronic kidney failure':ab,ti OR 'end-stage renal disease':ab,ti OR 'disease, end-stage renal':ab,ti OR 'end stage renal disease':ab,ti OR 'renal disease, end-stage':ab,ti OR 'renal disease, end stage':ab,ti OR 'renal failure, end-stage':ab,ti OR 'end-stage renal failure':ab,ti OR 'renal failure, end stage':ab,ti OR 'renal failure, chronic':ab,ti OR 'chronic renal failure':ab,ti OR esrd:ab,ti OR 'peritoneal dialysis, continuous ambulatory':ab,ti OR capd:ab,ti OR 'continuous ambulatory peritoneal dialysis':ab,ti

2.sacubitril:ab,ti AND 'valsartan sodium hydrate drug combination':ab,ti OR 'sacubitril valsartan sodium hydrate':ab,ti OR 'sacubitril-valsartan sodium hydrate drug combination':ab,ti OR (sacubitril:ab,ti AND 'valsartan drug combination':ab,ti) OR 'sacubitril valsartan drug combination':ab,ti OR 'sacubitril valsartan':ab,ti OR (sacubitril:ab,ti AND 'valsartan sodium anhydrous drug combination':ab,ti) OR 'sacubitril valsartan sodium anhydrous':ab,ti OR 'sacubitril-valsartan sodium anhydrous drug combination':ab,ti OR lcz696:ab,ti OR 'lcz 696':ab,ti OR entresto:ab,ti

**3.#1 AND #2**

Total79

Web of Science search strategy:

1.Renal Dialysis (Topic) or Dialyses, Renal (Topic) or Renal Dialyses (Topic) or Dialysis, Renal (Topic) or Hemodialysis (Topic) or Hemodialyses (Topic) or Dialysis, Extracorporeal (Topic) or Dialyses, Extracorporeal (Topic) or Extracorporeal Dialyses (Topic) or Extracorporeal Dialysis (Topic) or Hemofiltration (Topic) or Hemofiltrations (Topic) or Venovenous Hemofiltration (Topic) or Hemofiltration, Venovenous (Topic) or Venovenous Hemofiltrations (Topic) or Arteriovenous Hemofiltration (Topic) or Arteriovenous Hemofiltrations (Topic) or Hemofiltration, Arteriovenous (Topic) or Kidney Failure, Chronic (Topic) or End-Stage Kidney Disease (Topic) or Disease, End-Stage Kidney (Topic) or End Stage Kidney Disease (Topic) or Kidney Disease, End-Stage (Topic) or Chronic Kidney Failure (Topic) or End-Stage Renal Disease (Topic) or Disease, End-Stage Renal (Topic) or End Stage Renal Disease (Topic) or Renal Disease, End-Stage (Topic) or Renal Disease, End Stage (Topic) or Renal Failure, End-Stage (Topic) or End-Stage Renal Failure (Topic) or Renal Failure, End Stage (Topic) or Renal Failure, Chronic (Topic) or Chronic Renal Failure (Topic) or ESRD (Topic) or Peritoneal Dialysis, Continuous Ambulatory (Topic) or CAPD (Topic) or Continuous Ambulatory Peritoneal Dialysis (Topic)

1. sacubitril and valsartan sodium hydrate drug combination (Topic) or sacubitril valsartan sodium hydrate (Topic) or sacubitril-valsartan sodium hydrate drug combination (Topic) or sacubitril and valsartan drug combination (Topic) or sacubitril valsartan drug combination (Topic) or sacubitril-valsartan (Topic) or sacubitril and valsartan sodium anhydrous drug combination (Topic) or sacubitril valsartan sodium anhydrous (Topic) or sacubitril-valsartan sodium anhydrous drug combination (Topic) or LCZ 696 (Topic) or LCZ696 (Topic) or LCZ-696 (Topic) or Entresto (Topic)
2. **#1 AND #2**

Total 176

**Table S1.** Quality assessment of included studies according to the Newcastle-Ottawa Scale (NOS).

| First Author (Year) | Selection | Comparability | Outcome | Score/Maximum |
| --- | --- | --- | --- | --- |
| Chih-Yuan Niu2022 | ★★★ | ★★ | ★★★ | 8/9 |
| S-Lee2019 | ★★ | ★ | ★★★ | 6/9 |
| Bin Wang2021 | ★★★ | ★ | ★★★ | 7/9 |
| Zhonglin Feng2021 | ★★ | ★ | ★★★ | 6/9 |
| Ying Ding2023 | ★★ | ★★ | ★★★ | 7/9 |
| Sha Fu2021 | ★★ | ★ | ★★★ | 6/9 |
| J W Ma2023 | ★★ | ★★ | ★★★ | 7/9 |
| Cong Zhao2022 | ★★ | ★★ | ★★★ | 7/9 |
| Yanhong Guo2022 | ★★ | ★ | ★★★ | 6/9 |
| Sha Fu2023 | ★★★ | ★ | ★★★ | 7/9 |

A maximum of 4 stars for selection, 2 for comparability and 3 for the outcome.diameter;

**Table S2.** Blood pressure and Biomarkers before and after treatment of ARNI

| study | SBP(mmHg) | | DBP(mmHg) | | NT-proBNP (pg/ml) | |
| --- | --- | --- | --- | --- | --- | --- |
|  | Pre-ARNI | Post-ARNI | Pre-ARNI | Post-ARNI | Pre-ARNI | Post-ARNI |
| Chih-Yuan Niu | 136.0±19.0 | 132.0±20.9 | 72.5±13.8 | 70.8±14.4 | NR | NR |
| S-Lee | 109.4±45.7 | 114.7±24.3 | 82.9±25.2 | 76.7 ± 24.0 | NR | NR |
| Bin Wang | 161.6 ± 10.6 | 138.8±7.0 | 83.9 ± 8.2 | 74.8±9.7 | 9793.6±10723.6 | 5132.8±5889.6 |
| Zhonglin Feng | 143.3±18.1 | 141.3±19.6 | 78.6+12.1 | 77.6+13.7 | 31,239.2±8,742.5 | 20,315.1±12,933.6 |
| Ying Ding | NR | NR | NR | NR | 13575.8±18343.9 | 5757.5871±5814.6 |
| Sha Fu | 146.5±15.3 | 145.3±30.1 | 88.1±20.7 | 89.3±20.9 | 11743.8±14984.4 | 3726.8±3982.9 |
| J W Ma | 154±20 | 133±16 | 90±13 | 80±13 | 12114.1±13859.1 | 1023.0±936.2 |
| Fen Zhang | 144.6±8.5 | 138.6±7.6 | 91.9±5.4 | 89.5 ±5.4 | NR | NR |
| Cong Zhao | NR | NR | NR | NR | 40291.1±41619.3 | 13427.4±19624.6 |
| Yanhong Guo | 149.7 ± 23.6 | 137.2 ± 21.0 | 90.2 ± 16.1 | 84.5 ± 14.1 | 36757.0±42547.3 | 18344.9±25010.5 |
| Sha Fu | 157.8±20.5 | 141.5±16.8 | 85.2±14.9 | 78.5±10.2 | 9955.4±11103.5 | 5052.7±4448.2 |

ARNI, Angiotensin-receptor neprilysin inhibitor; SBP,systolic blood pressure；DBP，diastolic blood pressure ； NT-proBNP，N-terminal pro-B-type natriuretic peptide

**Table S3.** LV function indices after taking ARNI from baseline

| study | LVEF (%) | | LVESV (mL) | | LVEDV (mL) | | LVDd（mm） | | LVDs（mm） | |
| --- | --- | --- | --- | --- | --- | --- | --- | --- | --- | --- |
|  | Pre-ARNI | Post-ARNI | Pre-ARNI | Post-ARNI | Pre-ARNI | Post-ARNI | Pre-ARNI | Post-ARNI | Pre-ARNI | Post-ARNI |
| Chih-Yuan Niu | 31.3±5.5 | 45.1±11.7 | 95.7±33.3 | 70.1±35.7 | 140.00±43.6 | 123.9±36.1 | NR | NR | NR | NR |
| S-Lee | 30.2± 4.1 | 41.4± 10.5 | NR | NR | NR | NR | NR | NR | NR | NR |
| Bin Wang | 53.2 ± 5.9 | 56.3 ± 4.7 | 62.8 ± 15.2 | 53.3 ± 11.0 | 133.2 ± 25.9 | 122.6 ± 26.1 | 58.2±3.6 | 55.1 ± 4.7 | NR | NR |
| Zhonglin Feng | 38±5.25 | 45±10.0 | NR | NR | NR | NR | NR | NR | NR | NR |
| Ying Ding | 61.9±6.3 | 63.1±7.3 | NR | NR | NR | NR | 44.1±6.3 | 43.4±6.9 | 35.2±5.4 | 34.7±7.6 |
| Sha Fu2021 | 61.7±10.7 | 61.7±17.5 | NR | NR | NR | NR | NR | NR | 20.5±1.99 | 21.4±3.98 |
| J W Ma | 59.9±5.3 | 65.6±4.0 | NR | NR | NR | NR | 51.00±5.66 | 48.04±4.24 | NR | NR |
| Cong Zhao | 57.9±10.6 | 63.3±3.0 | 64.4±30.3 | 48.9±17.4 | 148.6±49.2 | 126.8±35.6 | 54±6.1 | 51.3±6.1 | 17.4±2.3 | 16.7±1.5) |
| Yanhong Guo | 61.4 ± 4.6 | 60.3 ± 6.7 | 61.0±29.5 | 53.6±26.8 | 142.8±46.6 | 132.8±43.3 | 53.8 ± 6.9 | 51.2 ± 7.1 | 17.2 ± 3.0 | 16.8 ± 2.8 |
| Sha Fu2023 | 57.0±10.3 | 58.7±8.1 | NR | NR | NR | NR | 46.0±7.8 | 47.8±6.2 | NR | NR |

ARNI, Angiotensin-receptor neprilysin inhibitor; LVEF, Left ventricular ejection fraction; LVEDV，left ventricular end- systolic volume ；LVEDV，left ventricular end-diastolic volume；LVDd，left ventricular end-diastolic diameter；LVDs，left ventricular end- systolic diameter NR, Not reported.

**Table S4.** LV function indices after taking ARNI from baseline

| study | E/e’ ratio | | Peak TR Vel(cm/sec) | | LAD（mm） | | IVSD(mm) | | LVPWT(mm) | |
| --- | --- | --- | --- | --- | --- | --- | --- | --- | --- | --- |
|  | Pre-ARNI | Post-ARNI | Pre-ARNI | Post-ARNI | Pre-ARNI | Post-ARNI | Pre-ARNI | Post-ARNI | Pre-ARNI | Post-ARNI |
| Chih-Yuan Niu | 25.3±16.2 | 18.8±6.4 | 272.9±78.7 | 234.2±65.4 | 46.9±7.2 | 43.7±8.1 | 11.5±2.9 | 11.4±2.6 | 10.9±2.0 | 11.2±2.7 |
| S-Lee | NR | NR | NR | NR | NR | NR | NR | NR | NR | NR |
| Bin Wang | 14.9 ± 5.8 | 12.9 ± 4.2 | NR | NR | NR | NR | 14.3 ± 2.1 | 14.0 ± 1.4 | NR | NR |
| Zhonglin Feng | NR | NR | NR | NR | NR | NR | NR | NR | NR | NR |
| Ying Ding | NR | NR | NR | NR | 44.1±6.3 | 43.4±6.9 | 11.4±2.4 | 11.4±2.3 | NR | NR |
| Sha Fu2021 | 29.4±37.9 | 20.8±21.0 | 269.9±89.2 | 244.9±81.1 | 37.7±4.0 | 38.0±4.8 | 11.6±2.4 | 12.0±3.2 | NR | NR |
| J W Ma | NR | NR | NR | NR | 41.97±5.18 | 38.85±4.41 | NR | NR | NR | NR |
| Cong Zhao | NR | NR | NR | NR | 41.4±6.8 | 37.3±9.1 | NR | NR | 11 .4±2.3 | 10.6±2.3 |
| Yanhong Guo | 8.9±4.0 | 7.4±2.1 | 280.5±52.2 | 240±59.7 | 40.5 ± 6.2 | 37.2 ± 7.2 | 11.8 ± 2.0 | 11.2 ± 2.0 | 11.8 ± 2.0 | 10.8 ± 1.9 |
| Sha Fu2023 | NR | NR | NR | NR | 37.5±5.7 | 38.7±6.1 | 12.8±1.9 | 12.9±2.0 | NR | NR |

ARNI, Angiotensin-receptor neprilysin inhibitor; E/e’ ratio，the ratio between early mitral inflow velocity and mitral annular early diastolic velocity; peak TR Vel ，peak tricuspid regurgitation velocity；LAD，left atrial dimension；LVPWT，left ventricular posterior wall thickness； IVSd， interventricular septum thickness in diastole

**Table S3.** LV function indices after taking ARNI from baseline

| study | E/e’ ratio | | Peak TR Vel(cm/sec) | | LAD（mm） | | IVSD(mm) | | LVPWT(mm) | |
| --- | --- | --- | --- | --- | --- | --- | --- | --- | --- | --- |
|  | Pre-ARNI | Post-ARNI | Pre-ARNI | Post-ARNI | Pre-ARNI | Post-ARNI | Pre-ARNI | Post-ARNI | Pre-ARNI | Post-ARNI |
| Chih-Yuan Niu | 25.3±16.2 | 18.8±6.4 | 272.9±78.7 | 234.2±65.4 | 46.9±7.2 | 43.7±8.1 | 11.5±2.9 | 11.4±2.6 | 10.9±2.0 | 11.2±2.7 |
| S-Lee | NR | NR | NR | NR | NR | NR | NR | NR | NR | NR |
| Bin Wang | 14.9 ± 5.8 | 12.9 ± 4.2 | NR | NR | NR | NR | 14.3 ± 2.1 | 14.0 ± 1.4 | NR | NR |
| Zhonglin Feng | NR | NR | NR | NR | NR | NR | NR | NR | NR | NR |
| Ying Ding | NR | NR | NR | NR | 44.1±6.3 | 43.4±6.9 | 11.4±2.4 | 11.4±2.3 | NR | NR |
| Sha Fu2021 | 29.4±37.9 | 20.8±21.0 | 269.9±89.2 | 244.9±81.1 | 37.7±4.0 | 38.0±4.8 | 11.6±2.4 | 12.0±3.2 | NR | NR |
| J W Ma | NR | NR | NR | NR | 41.97±5.18 | 38.85±4.41 | NR | NR | NR | NR |
| Cong Zhao | NR | NR | NR | NR | 41.4±6.8 | 37.3±9.1 | NR | NR | 11 .4±2.3 | 10.6±2.3 |
| Yanhong Guo | 8.9±4.0 | 7.4±2.1 | 280.5±52.2 | 240±59.7 | 40.5 ± 6.2 | 37.2 ± 7.2 | 11.8 ± 2.0 | 11.2 ± 2.0 | 11.8 ± 2.0 | 10.8 ± 1.9 |
| Sha Fu2023 | NR | NR | NR | NR | 37.5±5.7 | 38.7±6.1 | 12.8±1.9 | 12.9±2.0 | NR | NR |

ARNI, Angiotensin-receptor neprilysin inhibitor; E/e’ ratio，the ratio between early mitral inflow velocity and mitral annular early diastolic velocity; peak TR Vel ，peak tricuspid regurgitation velocity；LAD，left atrial dimension；LVPWT，left ventricular posterior wall thickness； IVSd， interventricular septum thickness in diastole

**Table S4.** effect of ARNI on cardiac function indices in contrast with ACEIs/ARBs

| study | LVEF, % | | LAd (mm) | | LVDd（mm） | | NT‑proBNP（ng/L） | | RVD, mm | | RAd, mm | |
| --- | --- | --- | --- | --- | --- | --- | --- | --- | --- | --- | --- | --- |
|  | ARNI | ACEIs/ARBs | ARNI | ACEIs/ARBs | ARNI | ACEIs/ARBs | ARNI | ACEIs/ARBs | ARNI | ACEIs/ARBs | ARNI | ACEIs/ARBs |
| Cong Zhao | 3.7±9.1 | 0.71 ± 6.15 | −3.65 ± 6.85 | −0.59 ± 5.71 | −2.72 ± 5.33 | −0.22 ± 7.01 | -20.09±31.59 | -8±27.17 | -0.35±3.78 | 0.65±2.30 | -1.65±9.84 | -0.06±9.99 |
| J W Ma | 5.7±4.78 | 1.4±4.42 | -3.12±4.84 | -1.82±5.67 | -2.96±5.10 | -1.8±3.82 | -11.09±13.42 | -3.08±6.98 | NR | NR | NR | NR |
| Ying Ding | 1.14±6.84 | -0.31±6.61 | -0.76±6.62 | -0.94±5.77 | NR | NR | NR | NR | -0.94±6.78 | -1.29±5.34 | -1.8±6.94 | -1.74±5.56 |

ARNI, Angiotensin-receptor neprilysin inhibitor ACEI,indicates angiotensin-converting enzyme inhibitor; ARB, angiotensin receptor blocker; LVEF, Left ventricular ejection fraction; LAD，left atrial dimension；LVDd，left ventricular end-diastolic diameter；NT-proBNP,N-terminal pro-B-type natriuretic peptide RVD, right ventricular diameter; RAD, right atrial diameter;

**Figure S1** Funnel plot estimating publication bias for changes of main parameters following ARNI. (A) systolic blood pressure (SBP)and diastolic blood pressure (DBP) (B) N-terminal proB-type natriuretic peptide（NT-proBNP) (C) Left Heart systolic function, (D) Left Heart diastolic Function, (E) and（F）LVEF

**Figure S2.** effect of ARNI on RAD and RVD compared with ACEIs/ARBs.

**Figure S3.** Subgroup analysis of ARNI effects on LVEF according to different proportions of patients with Duration of dialysis

**Figure S4.** Subgroup analysis of ARNI effects on LVEF according to different proportions of patients with follow-up periods.

**Figure S5.** Subgroup analysis of ARNI effects on SBP and DBP according to different proportions of patients with Study design

**Figure S6.** Subgroup analysis of ARNI effects on LVEF according to different proportions of patients with Study design

**Figure S7. Sensitivity analysis of the LVEF change.**

**Figure S8.** Correlation analysis of LVEF and LV function indices.(A) LVEF and LVESV; (B) LVEF and LVEDV; (C)LVEF and LVDd; (D) LVEF and LAD;（E）LVEF and IVSD;（F）LVEF an E/e’ ratio.
