# Supplementary figures and images for: Safety and efficacy of angiotensin receptor neprilysin inhibitor in improving cardiac function and blood pressure in dialysis patients
Source: Front Med (Lausanne). 2024 Sep 5;11:1421085. doi: 10.3389/fmed.2024.1421085 (PMC11410709; doi:10.3389/fmed.2024.1421085)

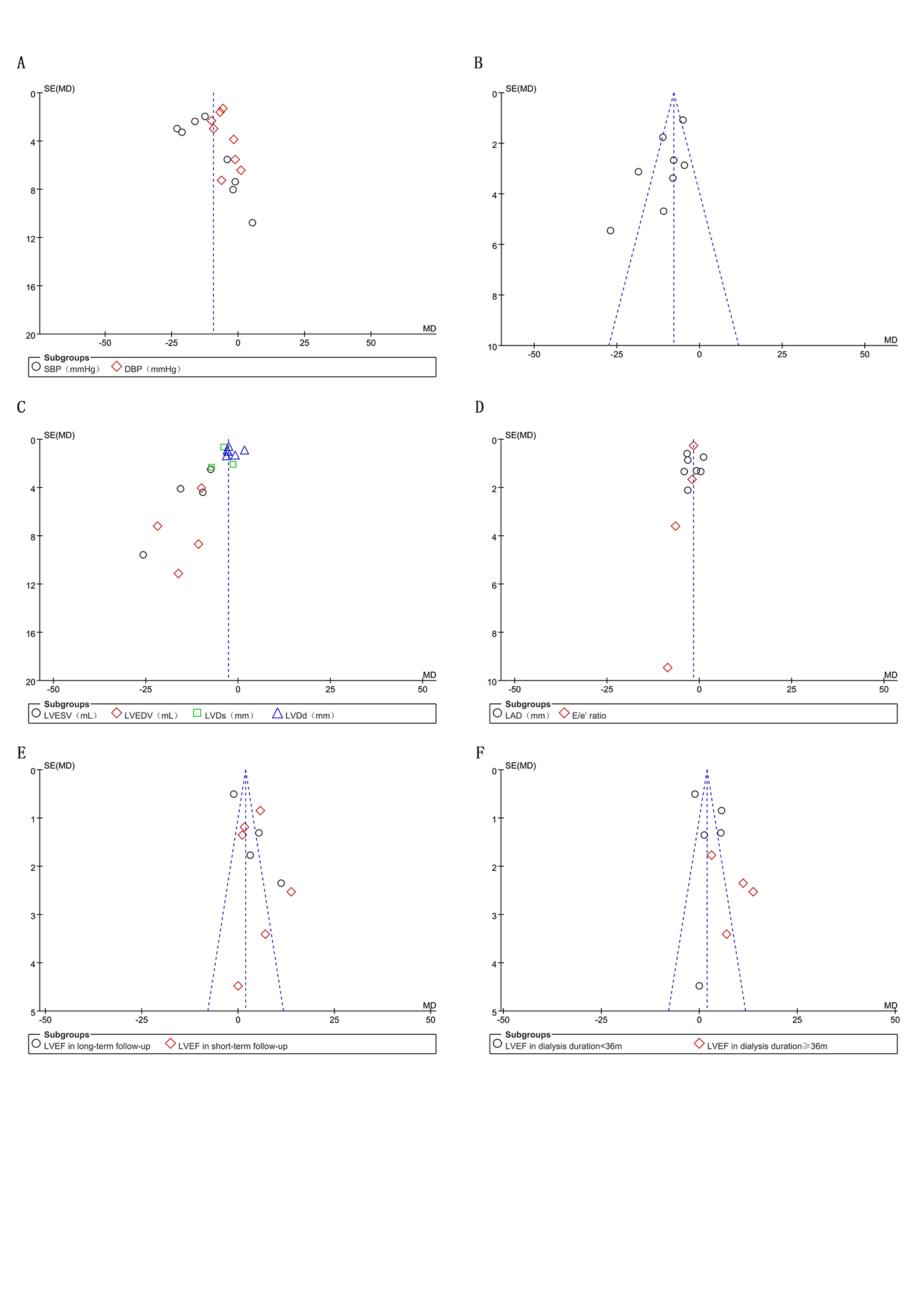

Supplement: Supplementary file 2 [file Image_1.tif]

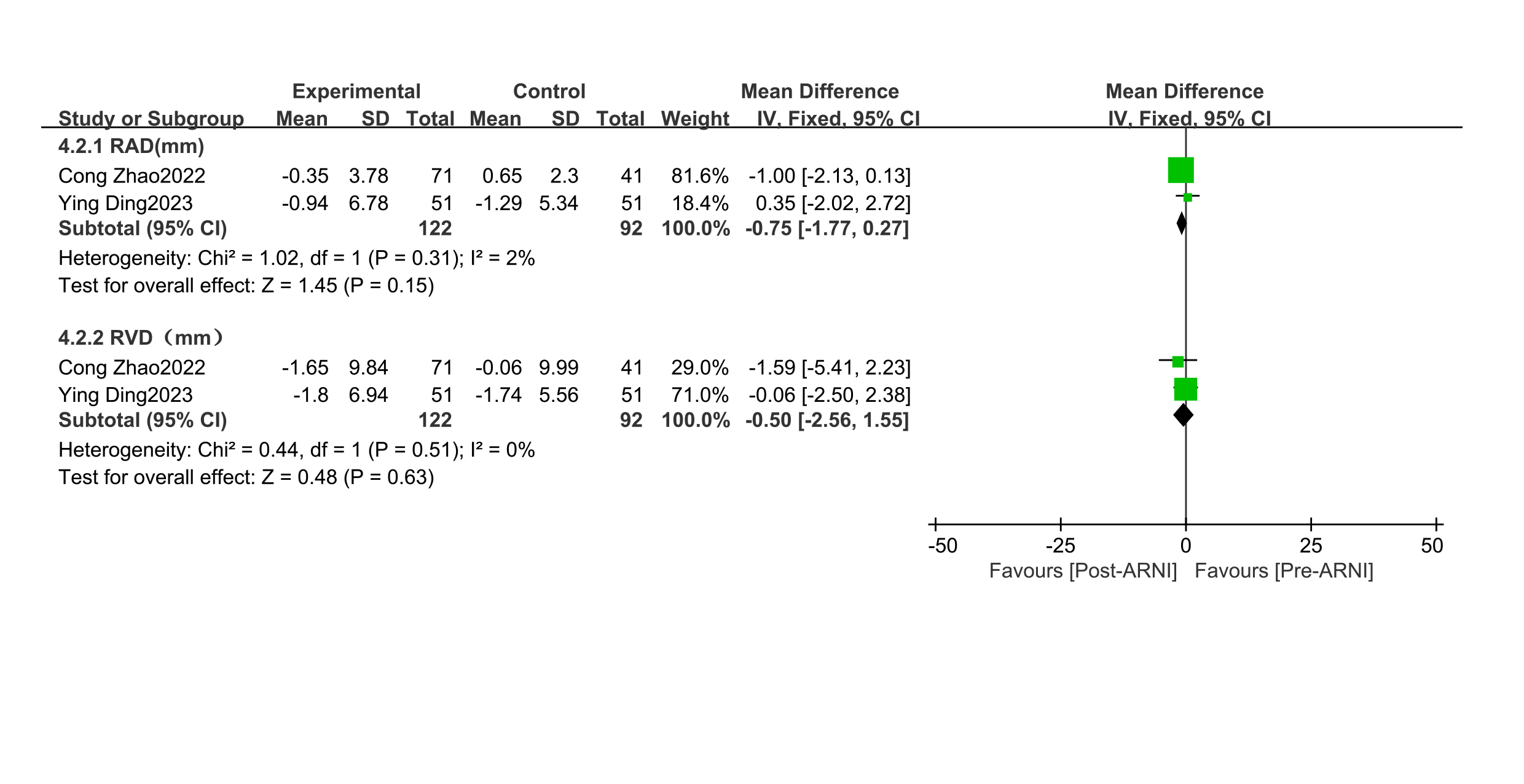

Supplement: Supplementary file 3 [file Image_2.tif]

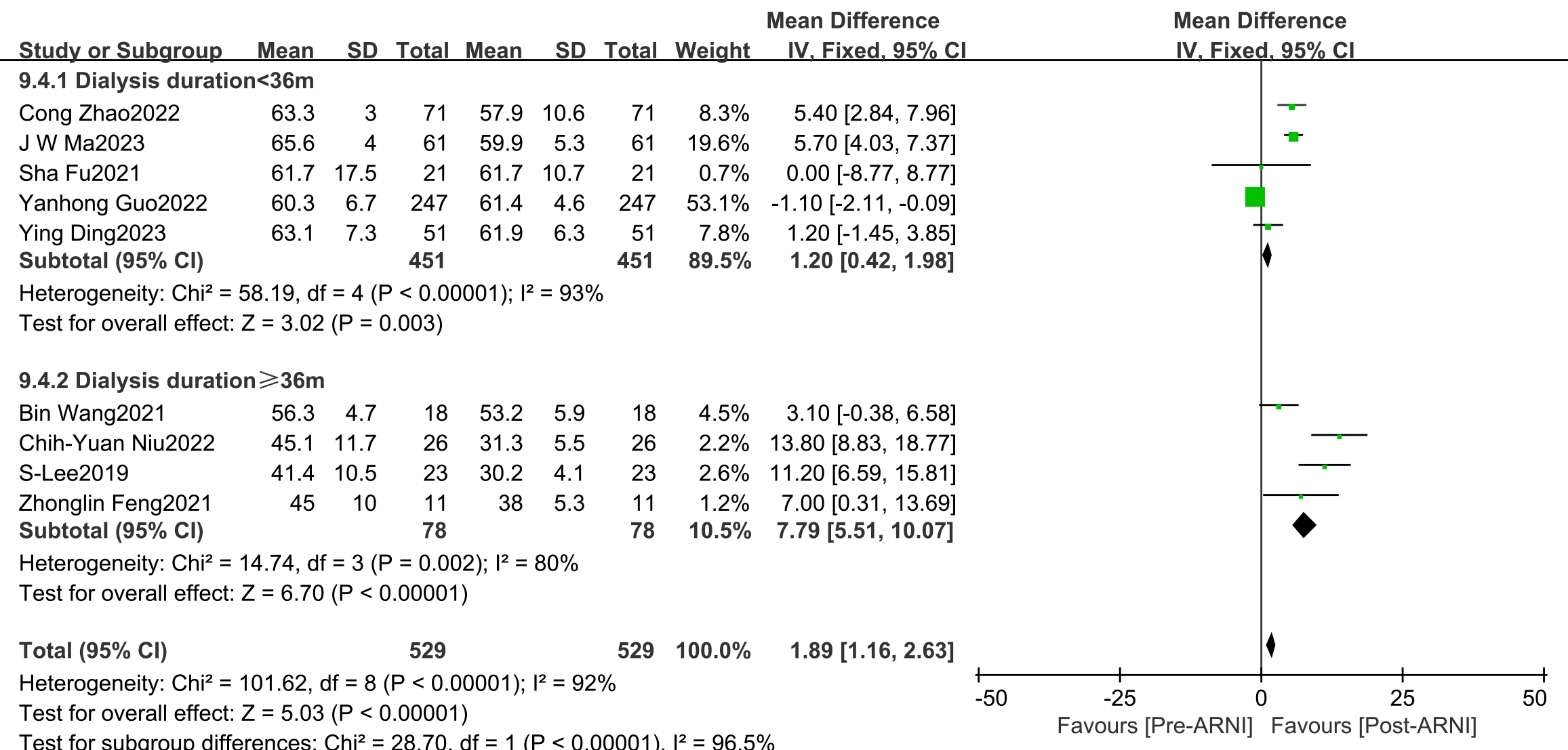

Supplement: Supplementary file 4 [file Image_3.tif]

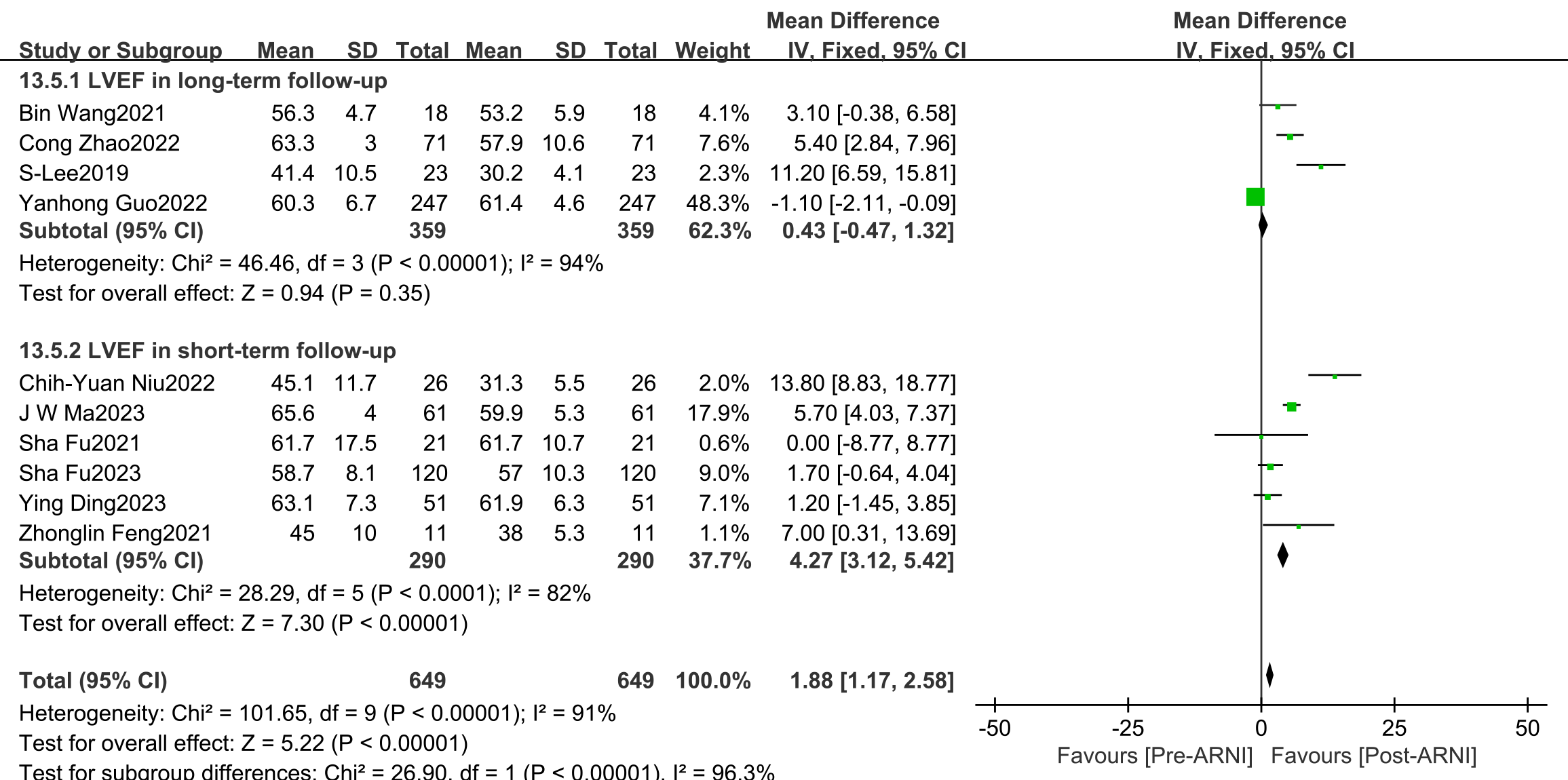

Supplement: Supplementary file 5 [file Image_4.tif]

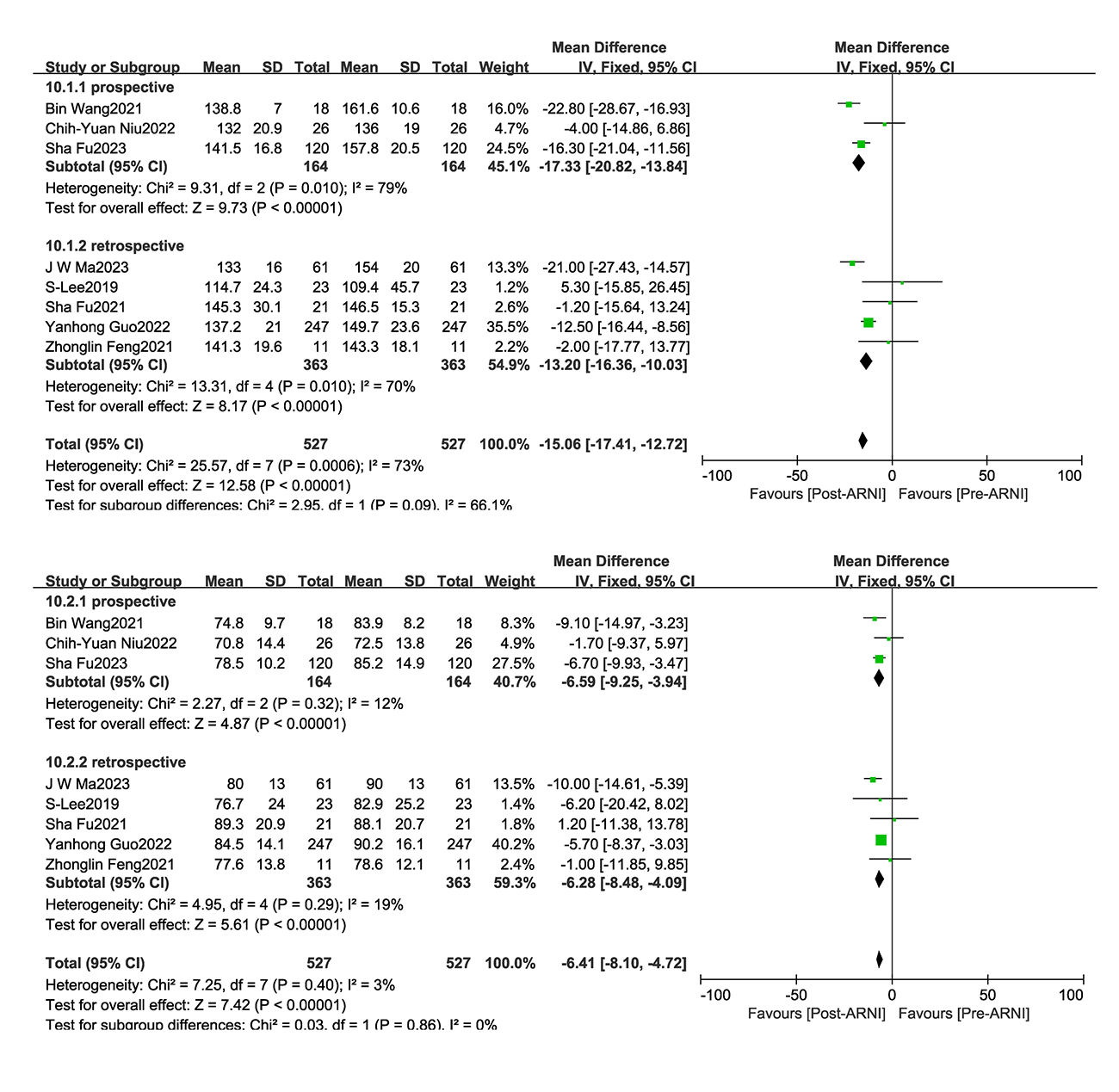

Supplement: Supplementary file 6 [file Image_5.tif]

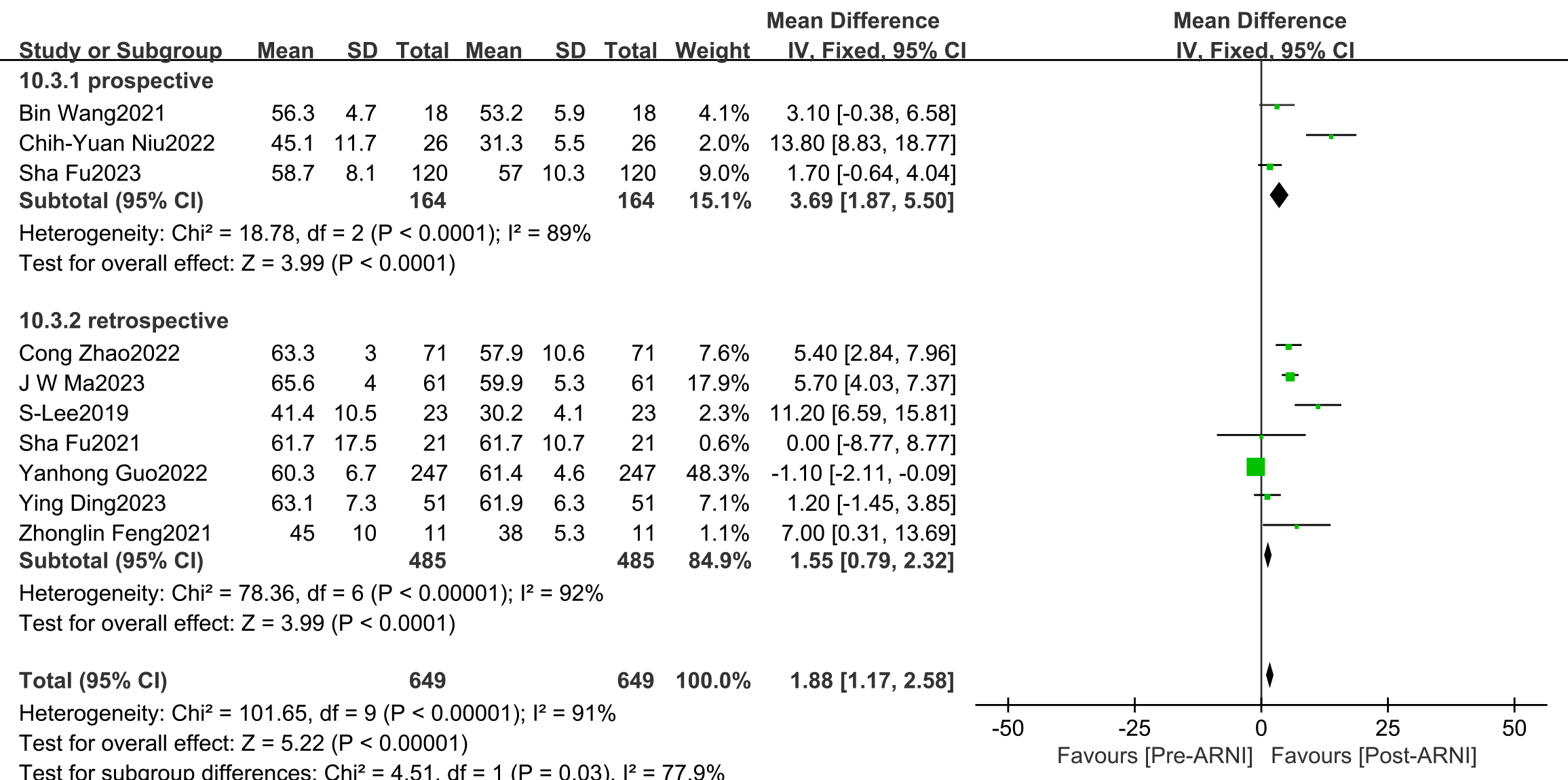

Supplement: Supplementary file 7 [file Image_6.tif]

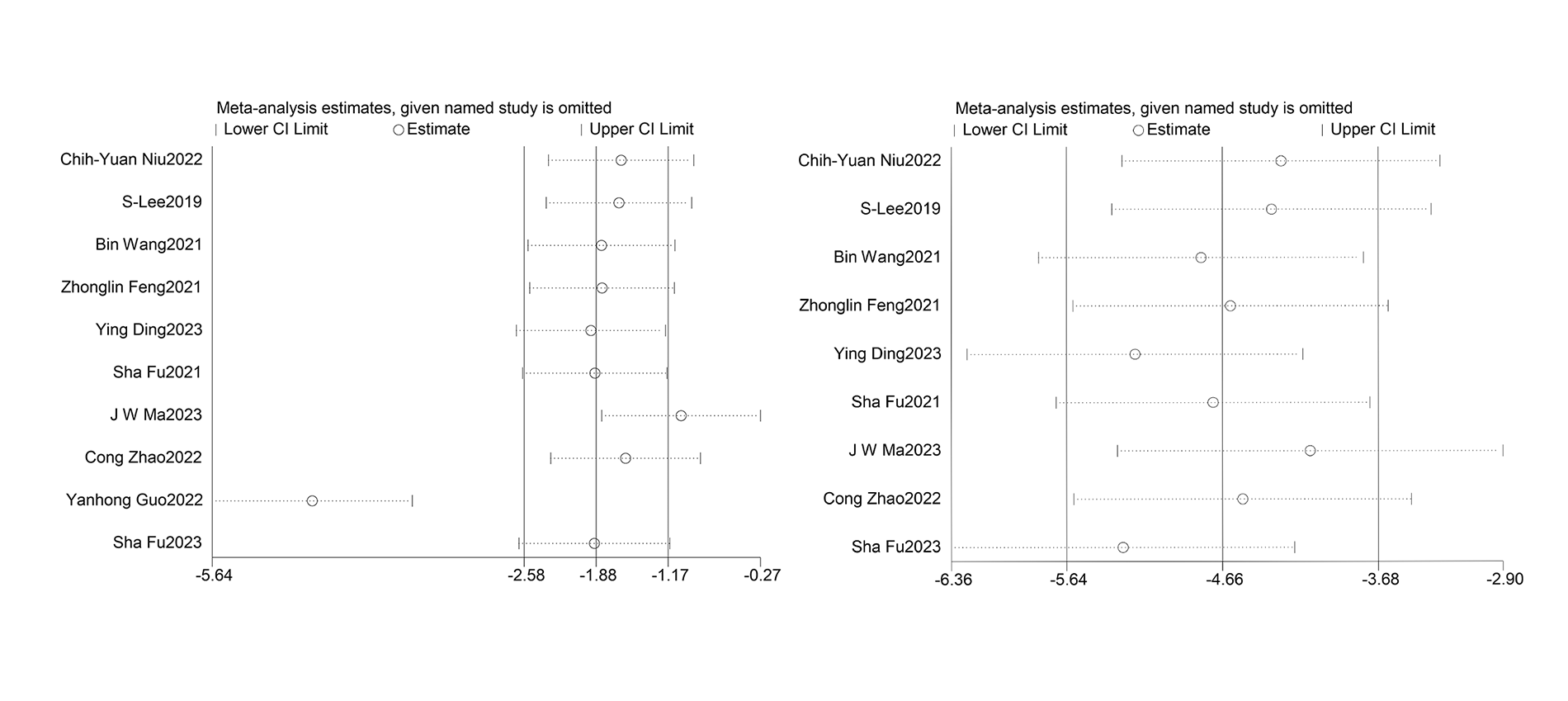

Supplement: Supplementary file 8 [file Image_7.tif]

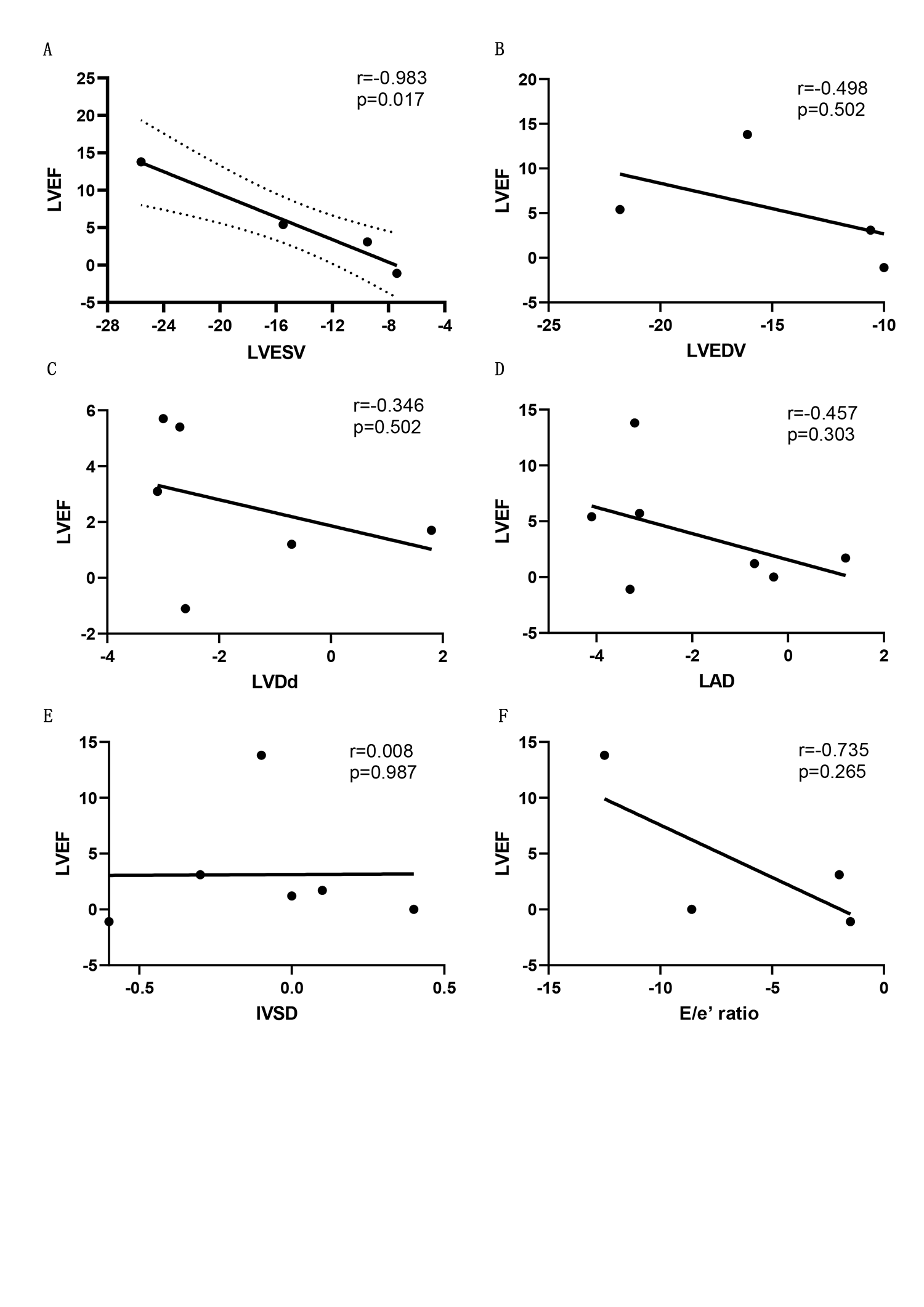

Supplement: Supplementary file 9 [file Image_8.tif]
